# NIR-Emitting Cyclometalated Cp*-Ir(III) Complexes: Impact of Ligand π‑Extension on Aggregation Behavior and Photophysical Properties

**DOI:** 10.1021/acs.inorgchem.5c05185

**Published:** 2026-01-30

**Authors:** Carlos Gonzalo-Navarro, María Rodríguez-Castillo, Miguel Monge, José M. López-de-Luzuriaga, Félix A. Jalón, Ana M. Rodríguez, M. Victoria Gomez, Gema Durá, Blanca R. Manzano

**Affiliations:** † 16733 Universidad de Castilla-La Mancha, Departamento de Química Inorgánica, Orgánica y Bioquímica- IRICA, Facultad de Ciencias y Tecnologías Químicas, Avda. C. J. Cela, 10, Ciudad Real 13071, Spain; ‡ Departamento de Química, Instituto de Investigación en Química (IQUR), 16764 Universidad de La Rioja, Madre de Dios 53, Logroño 26006, Spain; § Escuela Técnica Superior de Ingenieros Industriales, Avda. C. J. Cela, 3, Ciudad Real 13071, Spain

## Abstract

Half-sandwich iridium complexes exhibit poor photophysical properties. We reported the first case of half-sandwich cyclometalated iridium complexes with anticancer photodynamic activity, using two π-expansive ligands differing by one extra ring in [Cp*Ir­(C^N)*L*]­BF_4_ complexes. Considering the ability of the ligands to aggregate through π–π interactions, which may reduce the emission energy, and the interest in NIR emitters, we envisaged to study the concentration-dependent solution and solid state photophysical properties. Besides emission, aggregation was verified by ^1^H NMR, PFGSE-DOSY NMR experiments, X-ray diffraction, and DLS. It was found by X-ray diffraction the formation, with pair of enantiomers, of head-to-tail dimers that were further aggregated in some cases. The complexes with *L* = *N*-benzylimidazole became NIR emitters in solid state with a red-shift for the more π-expansive complex. The less π-expansive complex exhibited aggregation-enhanced emission. A significant effect on the emission of the additional ring was observed, that is explained by the stronger distortion of the excited state of the more π-expansive complex that favors disaggregation in solution. These are the first reported half-sandwich derivatives of any metal to exhibit NIR emission. DFT and TD-DFT studies support the experimentally observed features through the study of dinuclear model systems displaying π–π stacking interactions.

## Graphical abstract

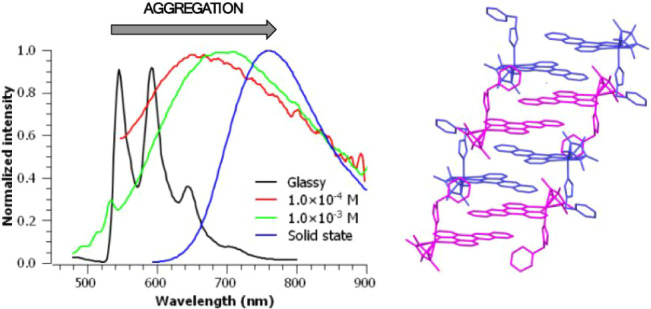

## Introduction

Half-sandwich iridium complexes usually exhibit poor photophysical properties, possibly because these compounds generate a relatively weak ligand field, even with C^N ligands, which places the nonradiative d–d (MC) state at an energy close to that of the emissive state. Accordingly, the use of half-sandwich iridium complexes in photodynamic therapy (PDT) is extremely rare. We recently reported the first example of half-sandwich cyclometalated iridium complexes exhibiting PDT activity.^1^ The strategy involved the use of ligands with extended π-systems to lower the energy of the ligand-centered (LC) ^3^ππ* state to below, or close to, that of the ^3^MLCT state, thereby increasing excited-state lifetimes. Two C^N ligands with different degrees of π extension were employed (see Chart [Fig chart1], pbpz and pbpn), and the results underscored the critical role of the additional fused ring present in the pbpn complexes. Only the complexes bearing the pbpn ligand, and not those containing the pbpz ligand, exhibited outstanding singlet oxygen (^1^O_2_) yields (up to 99%) and exceptional PDT performance.

**1 chart1:**
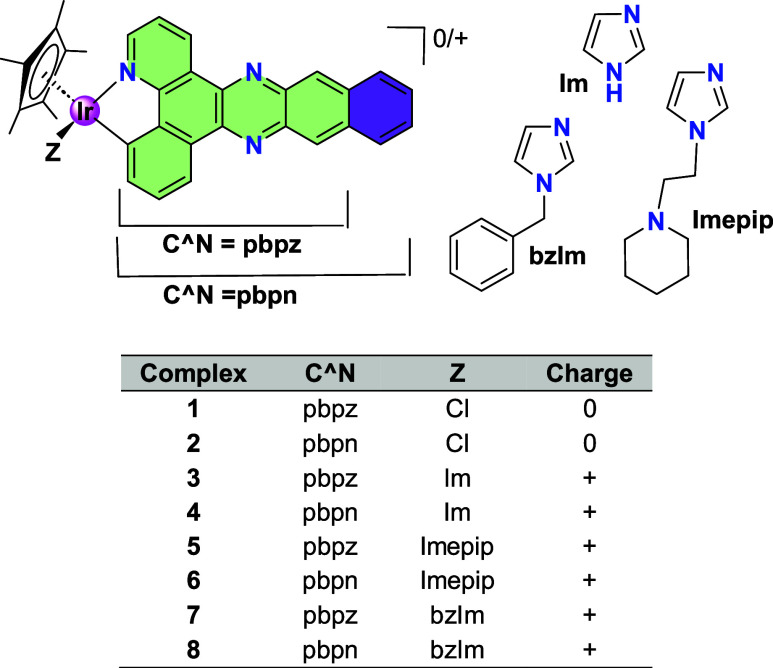
Half-Sandwich Ir­(III) Complexes^a^

a Cationic complexes **3**–**8** were isolated as tetrafluoroborate salts.

In view of the remarkable performance of these half-sandwich complexes and the π-extension of the ligands, we undertook a more in-depth investigation of their photophysical properties in both solution and the solid state, as well as the potential influence of complex aggregation on these properties. The impact of the presence or absence of the additional fused ring on these properties was also examined. It was anticipated that aggregation could induce a red shift in the emission. This aspect is particularly relevant given the widespread use of near-infrared (NIR) emitters in applications such as telecommunication networks, night vision,^2^ security authentication,^3^ organic light-emitting diodes (OLEDs),^4–6^ light-emitting electrochemical cells (LECs)^4,7,8^ bioimaging,^9,10^ and photodynamic therapy.^10,11^ Solid-state NIR light-emitting electrochemical cells (LECs) have demonstrated several advantages over NIR organic light-emitting devices (OLEDs), including low-voltage operation and the avoidance of the more complex fabrication processes required for OLEDs.^12,13^ However, the development of NIR emitters that operate efficiently in the solid state remains particularly challenging because of aggregation-caused quenching effect (ACQ).^14^

We were aware that, in our case, ACQ could be operative and that narrowing of the energy gap might lead to a reduced photoluminescence quantum yield. As the energy of the emissive excited state decreases, efficiencies typically decline due to the combined effects of enhanced nonradiative decay pathways and reduced oscillator strengths.^6^ In any case, aggregation-enhanced emission (AEE) remained a plausible alternative process.^15^

In this paper we describe a detailed study of the photophysical properties of the complexes [Cp*Ir­(C^N)­(bzIm)]­BF_4_, with C^N = pbpz, **7**, and pbpn, **8**, bzIm = *N*-benzylimidazole, both in solution, in glassy solution and solid state and the possible influence on these properties of aggregation. Various complementary techniques, such as ^1^H NMR, DOSY, X-ray diffraction, UV-vis absorption and emission, DLS (dynamic light scattering) have been applied in this study, including, in some cases, other related complexes.

Significant differences have been observed between **7** and **8**, attributed to the extra fused ring in **8**. In the structures of complexes with pbpz or pbpn, as determined by X-ray diffraction, head-to-tail dimers are formed from pairs of enantiomers. These dimers further aggregate in different ways. **7** and **8** become NIR emitters in solid state with a red-shift in the case of **8**. Interestingly, **7**, and not **8**, exhibits a significant increase in the photoluminescent quantum yield when the concentration is increased and in solid state, displaying aggregation enhanced emission (AEE). Furthermore, **7** and **8** are the first reported half-sandwich metal complexes that behave as NIR emitters. DFT and TD-DFT studies support the experimentally observed features of these complexes through the study of dinuclear model systems displaying π–π stacking interactions, which are of paramount importance for the description of their NIR-emissive behavior.

## Results and Discussion

### Ir­(III) Half-Sandwich Complexes

These compounds were previously prepared by us, by the cyclometalation of the C^N ligand and the subsequently coordination of the imidazolyl-derivatives after chloride removal in the presence of a silver salt. Their purity was determined by HPLC analysis and all of them were fully characterized by NMR spectroscopy, MS spectroscopy and elemental analysis.^1^ The general formulas of complexes are [Cp*Ir­(C^N)­Cl] and [Cp*Ir­(C^N)*L*]­BF_4_ (C^N = pbpz, pbpn; *L* = imidazolyl -derivatives; Chart [Fig chart1]). Complexes **7** and **8** were selected as representative examples (**7**: C^N = pbpz, **8**: C^N = pbpn; *L* = *N*-benzylimidazole, bzIm) to study the photophysical properties in depth. Complexes **3** and **7** were also characterized by X-ray diffraction.

### X-ray Crystal Structures

The molecular and crystal structures of complexes **3** and **7 × 0.75C**_**3**_ **H**_**6**_ **O** were determined by X-ray diffraction. The X-ray crystal structures of complexes **1** and cation **6 × 0.75C**_**3**_ **H**_**6**_ **O** were previously described by us.^1^ The complexes crystallize in the space group *P*2_1_/*n* of the monoclinic system (**3**) or in *PI̅* of the triclinic system (**7**). The crystallographic data are provided in Table S1 and selected bond distances and angles are collected in Table S2. The corresponding ORTEP diagrams are shown in Figure 1. The unit cells of the complexes contain both enantiomers, *R*_Ir_ and *S*_Ir_, arising from the stereogenic nature of the metal center. In the case of **7**, four molecules are present in each asymmetric unit.

**Figure 1:**
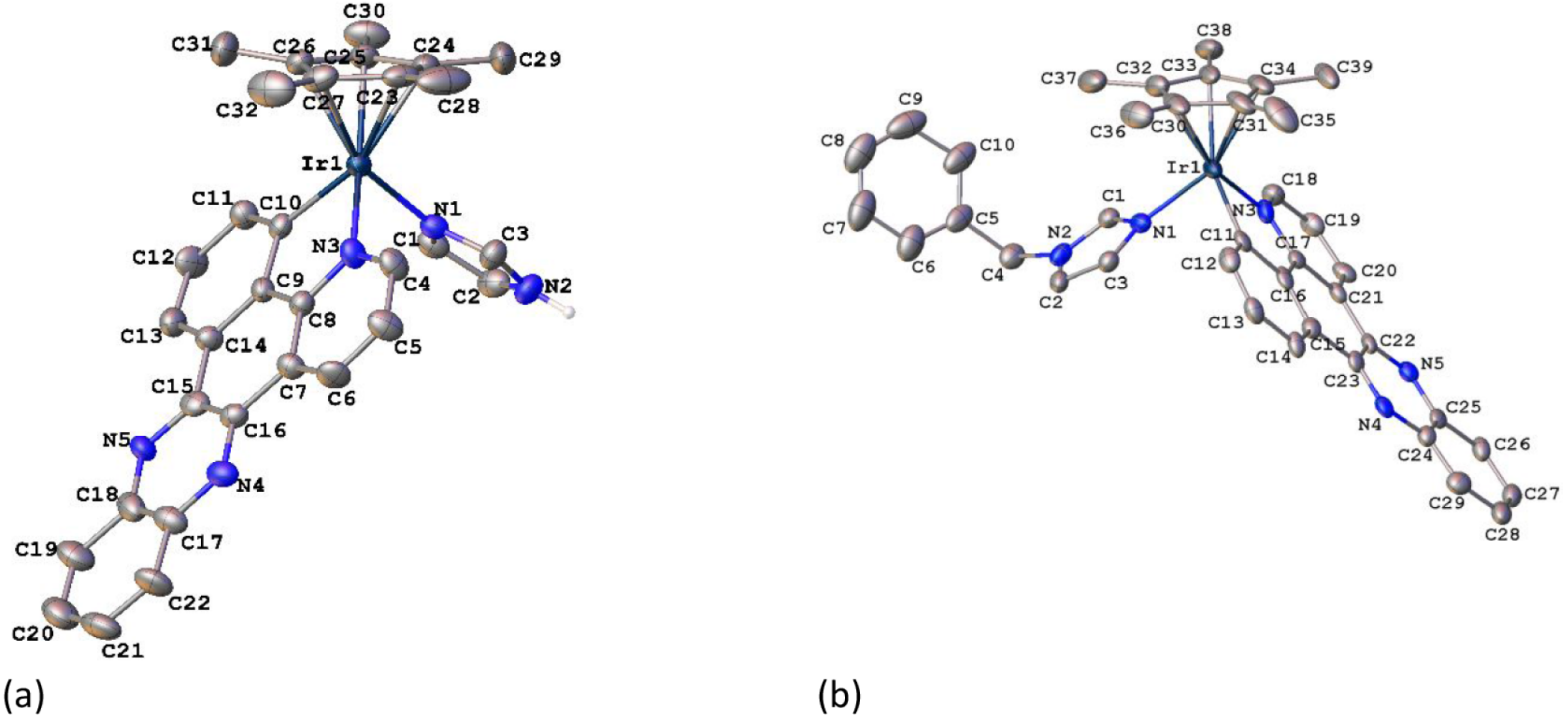
ORTEP diagrams of cations of *R*_Ir_ **3** (a) and *R*_Ir_ **7** (b). The orientation of the benzyl fragment shown for **7** corresponds to that of three molecules of the asymmetric unit (with Ir1–Ir3). The orientation of the fourth molecule is shown in the SI (Figure S1). Ellipsoids are at the 30% probability level. Hydrogen atoms and BF_4_^–^ anions have been omitted for clarity.

The complexes exhibit the expected pseudo-octahedral half-sandwich geometry with the Cp* ring adopting an η^5^-coordination mode and occupying three coordination sites, while the C^N ligand exhibits a bidentate-chelate coordination mode (κ^2^-*C,N*). An imidazole ring (**3**) or a benzyl-imidazole ring (**7**) complete the coordination sphere around the metal center. The distances involving the Ir atom are typical distances of this type of compound.^1,16,17^

The π-extended ligands retain their planarity upon coordination to the metal atom. The dihedral angle formed by the plane of the two coordinated rings and that of the quinoxaline fragment is 1.48° in **3** and in the range 1.44–5.55° for the four molecules of **7**. Some variation is observed in the Ir–C (in the range 2.04–2.09 Å) or Ir–N (from 2.02 to 2.10 Å) distances. Considering the Cp* ring, the Ir–centroid distances vary in the range 1.81 and 1.82 Å, with similar values to those reported in the literature.^16,17^ The two longest Ir–C distances are those situated approximately *trans* to the Ir–C­(C^N) bond, due to the higher *trans* influence of the C-donor ring, in a similar way to cases described with other non-π-extended C^N ligands.^18^ There are small differences in the bite angle of the C^N ligand (78.3–79.3°). The dihedral angle formed between the plane of the imidazolyl ring and that of the C^N ligand varies between 79.0° (molecule of Ir-2 in **7**) and 89.5° in **3**. In **7**, the *N*-benzyl fragment for cations with Ir-1 to Ir-3 is oriented in the opposite direction to the C^N ligand (see Figure 1). The arrangement is reversed in the case of the Ir-4 cation (Figure S1). We propose that these orientations are determined by packing forces.

Concerning noncovalent interactions, the previously described^1^ complexes **1** and **6** will also be considered for comparison. It is to note that **6** contains the pbpn ligand. As expected, all the derivatives exhibit π–π interactions involving the C^N ligands. Other examples of π–π interactions in cationic complexes involving an N^N ligand similar to pbpz have been reported.^19–22^ The four complexes exhibit π–π interactions between the two enantiomers in a head-to-tail disposition, with shorter centroid–centroid distances and a higher number of interactions observed for complex **6**, which has the more π-expansive ligand. The π–π interactions involve the quinoxaline (**1**, **3** and **7**) or benzoquinoxaline (**6**) fragment plus the contiguous ring. The centroid–centroid distances are shown in Figures S2–S5. In the three cationic derivatives box-like dimers are formed through the cooperativity between CH−π (imidazolyl ring) and π–π interactions (see parameters in Tables S3 and S4) (hydrogen bonds with BF_4_^–^ are also present in **6**). In Figure 2 the dimers of one complex with pbpn (**6**) or pbpz (**7**) ligands are reflected. This disposition of the two cations is reminiscent of the quadruple pyrazolyl^23–25^ or imidazolyl^26,27^ embrace, where four rings are connected by one π–π and two CH−π interactions. Other examples involving other rings have been reported^28^ but the ones reported here are special as they involve 8 or 10 rings. It is worthy of note that the averaged value of the Ct–Ct (Ct = centroid) distances is smaller in **6** (3.72 Å), with the more π-extended ligand (the values in the pbpz complexes are in between 3.79 and 3.88 Å). The dimer of **6** also contains the highest number of π–π interactions. In the case of **7**, other π–π interactions are also present involving rotation (57.9° and 122.1°) of the two interacting units (Figure S6) which originates infinite polymeric chains (Figure S7). However, there are not π–π interactions between the dimers in the case of **6.** In Figure 3 the differences in the packaging of dimers of complexes **6** and **7** are reflected.

**Figure 2:**
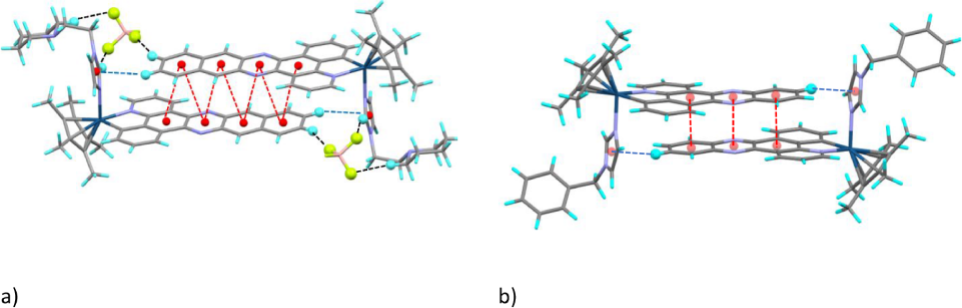
Box-like dimers of complex **6** (a) or **7**(Ir1) (b) formed through π–π interactions (red), CH−π interactions (blue) and hydrogen bonds with the BF_4_^–^ anions (black). The H and F atoms that participate in H bonds or CH−π interactions are marked as balls.

**Figure 3:**
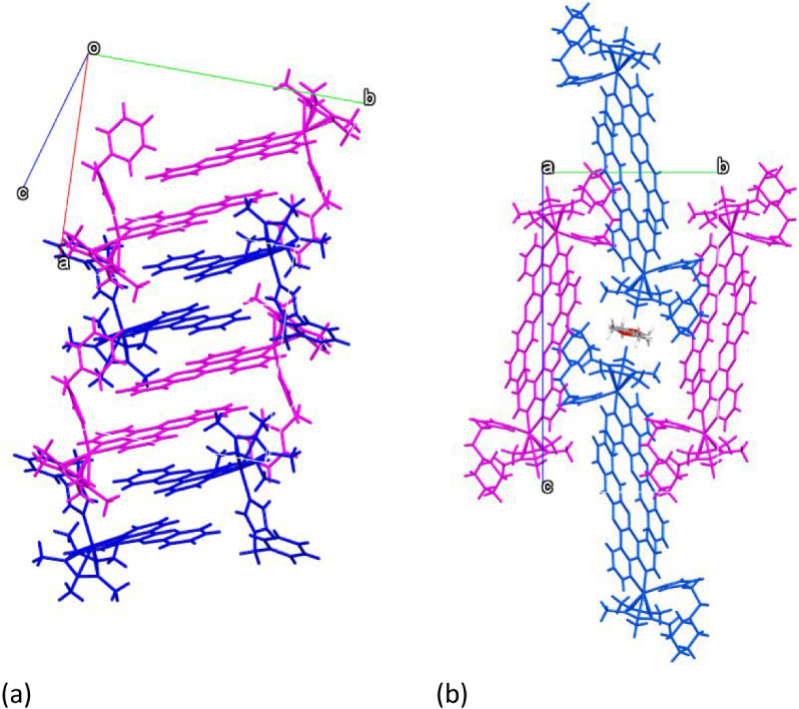
Dimer packaging of **7** (a) and **6** (b). The two molecules that combine to form a dimer share the same color.

### Analysis of π–π Stacking by ^1^NMR Spectroscopy and DOSY Studies

Given the π-expansive nature of the C^N ligands and the π–π stacking interactions observed in the solid state, we decided to ascertain whether this interaction also occurs in solution. Therefore, the complexes were studied by ^1^H NMR spectroscopy at different concentrations. This study examined complexes **1–8** to determine the effect on the stacking of the nature of the C^N or *L* ligand. An increase in concentration could reveal the presence of π–π stacking interactions through shielding of the ring proton resonances due to the influence of the ring current from adjacent aromatic moieties.^29,30^ Additionally, this study could provide insight into the regions primarily involved in the interaction.

The differences in chemical shifts of the C^N aromatic protons for the most dilute and concentrated solutions in CDCl_3_ or (CD_3_)_2_CO are provided in Table 1. The corresponding set of spectra are given in Figures S8–S15. Although all C^N aromatic protons exhibit concentration-dependent changes in chemical shift, the magnitude of the shielding effect upon increasing concentration depends on the specific compound. The Δδ values are lower for the chlorido complexes; however, the use of chloroform—necessitated by solubility constraints and possessing lower polarity than acetone—may influence these trends. When comparing complexes with the same *L* ligand, higher Δδ values are observed for the pbpn complexes containing the more π-expansive ligand, likely reflecting a more efficient π-stacking interaction. Among the pbpn complexes bearing imidazolyl-derived *L* ligands, the strongest shielding is observed for complex **4**, which contains the imidazole ligand. Analysis of the individual proton responses reveals that, for the three cationic pbpn complexes, the Δδ values follow the order H^4^, H^19^, H^20^ > H^10^ > H^23^, H^24^, H^25^, H^26^ > H^5^, H^11^ > H^6^, H^12^. This trend is consistent with a head-to-tail aggregation mode, as observed in the solid state, in which the protons closest to the metal center are least affected. A similar conclusion was reached for the corresponding pbpz derivatives. In contrast, the protons of Cp* and *L* ligands are essentially insensitive to changes in concentration. Figure 4 illustrates the two general structures, with blue circles whose sizes are approximately proportional to the corresponding Δδ values.

**Table 1:** Effect of the Concentration on the ^1^H NMR Resonances, Δ­(δ_dil_ – δ_conc_), for **1** and **2** in Chloroform-*d_3_* from 3 mM to 24 mM and for **3**–**8** in Acetone-*d_6_* from 2.5 to 25 mM^a^

		Δδ (ppm)									
Comp.	C^N	H^4^	H^5^	H^6^	H^10^	H^11^	H^12^	H^19^/H^20^	H^21^/H^22^	H^23^/H^24^	H^25^/H^26^
**1**	**pbpz**	0.04	0.02	0.01	**0.10**	0.02	0.03	**0.13**/0.07	0.04	–	–
**2**	**pbpn**	0.07	0.05	0.02	0.08	0.03	0.03	**0.20**/**0.10**	–	0.05	0.05
**3**	**pbpz**	0.02	0.03	0.00	0.02	0.01	0.01	0.02	0.03	–	–
**4**	**pbpn**	**0.26**	0.08	0.03	**0.19**	0.07	0.02	**0.26**	–	**0.18**	**0.18**
**5**	**pbpz**	0.07	0.04	0.01	0.05	0.02	0.01	0.05	0.04	–	–
**6**	**pbpn**	**0.20**	0.06	0.01	**0.14**	0.05	0.01	**0.18**	–	**0.11**	**0.10**
**7**	**pbpz**	**0.11**	0.08	0.00	**0.10**	0.02	0.01	0.10	0.05	–	–
**8**	**pbpn**	**0.22**	0.06	0.02	**0.18**	0.06	0.02	**0.22**	–	**0.15**	**0.15**

a Values of Δδ ≥ 0.10 are highlighted in bold.

**Figure 4:**
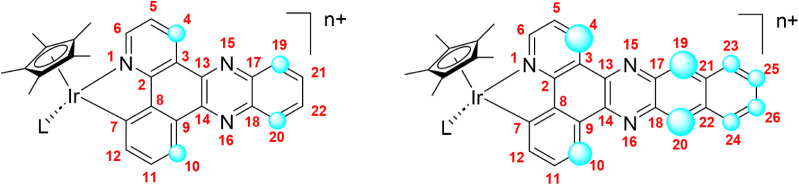
General structures for complexes with pbpz (left) or pbpn (right) C^N ligands. The blue circles reflect in an approximate way the Δδ values.

Complexes **7** and **8**, both exhibiting clear Δδ values but with notable differences between them, were selected for more detailed investigation. Solution aggregation was examined in greater detail by ^1^H NMR spectroscopy over a broad concentration range (0.025–25 mM, Table S5). Figure 5 shows the concentration-dependent variation of the δ values for the H^4^ and H^10^ protons. At the highest concentration, a more pronounced reduction in chemical shift is observed for the complex bearing the more π-expansive ligand (**8**).

**Figure 5:**
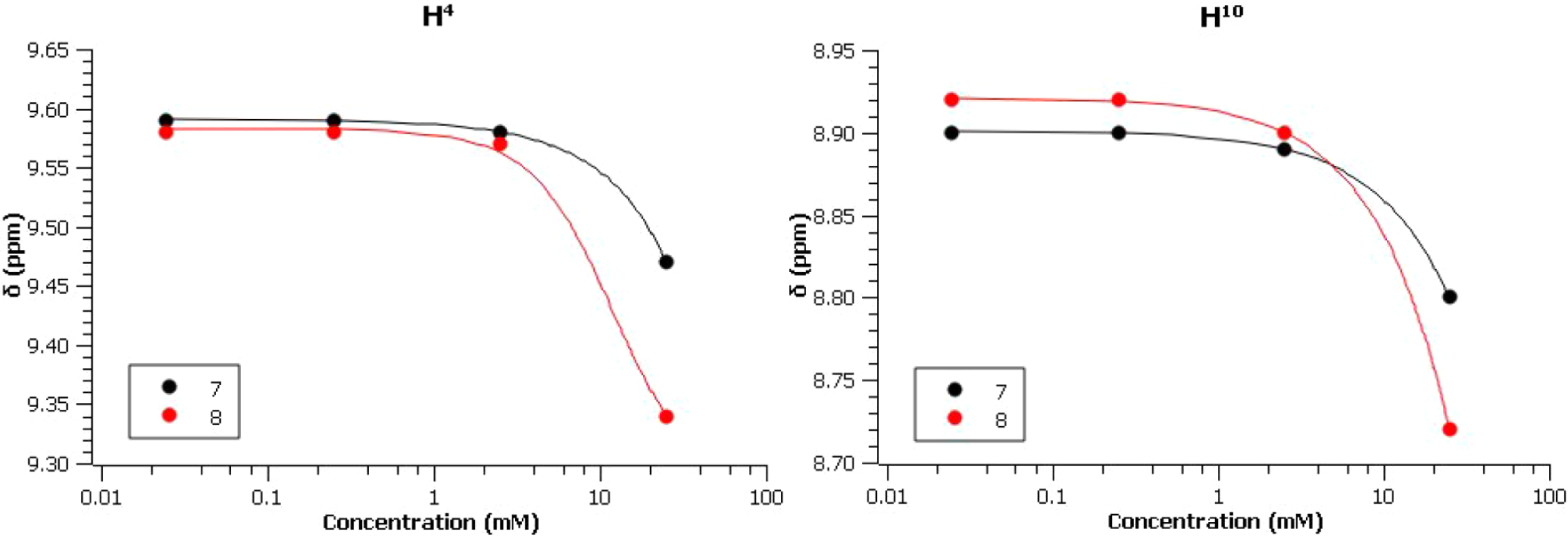
Observed chemical shifts (δ) of protons H^4^ and H^10^ in the ^1^H NMR spectra of complexes **7** and **8** as a function of the concentration in acetone-*d*_6_.

Focused on gaining a better insight into the behavior of compounds **7** and **8** in solution, ^1^H pulsed field-gradient spin echo (PFGSE) NMR experiments (DOSY NMR experiments) were carried out in acetone. The translational self-diffusion coefficients (*D*_t_) can be accurately evaluated by PFGSE NMR spectroscopy experiments, providing, in itself, important information about the tendency of a compound to aggregate in solution.^31^ The differences in chemical shift variation observed in concentration-dependent ^1^H NMR experiments explained above are consistent with the results observed in the PFGSE experiments, with the pbpn complexes with imidazolyl-derived ligands *L* showing the strongest changes. Thus, a concentration range of 2–25 mM was selected, with the lower limit determined by the detection threshold of the NMR spectrometer and the upper limit constrained by the solubility of the compounds in acetone. A reference (in our case TMS) was used as an internal standard to compensate for viscosity changes among solutions of different concentrations as previously reported.^32^ In this way, changes in *D* values can be confidently attributed to aggregation phenomena rather than to variations in viscosity in concentration-dependent NMR experiments. Thus, for each sample concentration, two PFGSE experiments were performed, focusing on the signal attenuation as a function of the gradient strength for both, the Cp* resonance (1.8 ppm) of the compound of interest (**7** or **8**) and the TMS signal. Considering that one of the main problems associated with the diffusion experiments is the anomalous large diffusion constants produced by thermal convection currents, the experiments were performed without temperature control to minimize these effects.

^1^H DOSY indicates that compound **8** exhibits a clear decrease in the translational diffusion coefficient when going from the lower to the higher concentrations (see Figures S16 and S17) (entries 1–3, column 4, Table 2 and Table S7). In contrast, compound **7** (entries 1–3, column 3, Table 2, Table S7) does not show such a tendency as also observed in Figures S18 and S19, where the Log*D*_t (**7**)_ values for the three different concentrations can not be differentiated. A more accurate and robust comparison, which minimizes the effects of viscosity variations due to differences in concentration or temperature, can be achieved by evaluating the ratio *D*_t (compound)_/*D*_t (TMS)_.^32^ Compound **7** shows a 3% variation (entry 3 versus entry 1, column 3, Table S6) whereas compound **8** exhibits a 16% variation (entry 3 versus entry 1, column 4, Table S6), which again evidence the higher aggregation propensity of compound **8** relative to compound **7** in acetone over the studied concentration range. Table S7 shows all diffusion coefficients including the standard deviation for every sample.

**Table 2:** Concentration (*C*, mM) Diffusion Coefficients (Log*D*_t_, m^2^ s^–1^) for Complex **7** and **8** at RT in Acetone

Entry	Concentration (mM)	Log*D*_t (**7**)_	Log*D*_t (8)_
**1**	2	–9.36	–9.34
**2**	12	–9.34	–9.42
**3**	25	–9.38	–9.46

### Dynamic Light Scattering (DLS) Studies

DLS analyses were carried out in DMSO:H_2_O (1:9) for complexes **7** and **8** to evaluate their aggregation behavior at two distinct concentrations (Table 3; Figures S20–S21 for the corresponding histograms). This solvent system, predominantly aqueous, differs substantially from those employed in NMR or photophysical investigations. Owing to its high polarity, water is anticipated to promote pronounced aggregation in these complexes featuring extensive hydrophobic domains.

**Table 3:** DLS Diameter for Complexes **7** and **8**, at Different Times and Concentrations

Com.	Conc. (μM)	0 min	15 min	1 h	4 h
**7**	50	188	192	227	239
	250	206	212	215	219
**8**	25	129	127	128	131
	250	118	114	116	120

Complex **7** exhibited aggregation at 50 μM, forming particles with an average diameter of ∼188 nm; no stable aggregates were detected below this concentration. Over time, aggregate size increased slightly to ∼239 nm. At 250 μM, aggregates were marginally larger than at the lower concentration but remained stable after 4 h.

Complex **8** aggregated at a minimum concentration of 25 μM, yielding particles of ∼129 nm that persisted unchanged after 4 h. At 250 μM, aggregates measured ∼118 nm and were similarly stable over time.

These findings confirm that both complexes form stable aggregates, with complex **7** producing larger assemblies, whereas complex **8** aggregates at lower concentrations. Although notable differences exist between the solvent systems, this last observation aligns with the more pronounced π-stacking interactions identified for the dimers in the pbpn complex by X-ray diffraction. Furthermore, it is consistent with studies reporting concentration-dependent variations in ^1^H NMR chemical shifts, supported by complementary DOSY analyses.

### Photophysical Properties

The photophysical properties of complexes **7** and **8** were comprehensively investigated both in solution and in the solid state. Their absorption, emission, and other relevant photophysical parameters are summarized in Table 4 (solution) and Table 5 (solid state). In solution, the low-energy region of the absorption spectra, attributed to the quinoxaline units of [Cp*Ir­(pbpn)­(*L*)]^+^ complexes, is red-shifted by approximately 50 nm relative to the corresponding region in [Cp*Ir­(pbpz)­(*L*)]^+^ complexes (Figure 6a). This shift is likely associated with the stronger electron-withdrawing character of the pbpn ligand compared to the pbpz ligand, due to higher π-conjugation, as discussed in our previous work, and is assigned to a mixture of ^1^MLCT (d→π*) and ^1^LC (π→π*) spin-allowed transitions.^1^ Furthermore, in both complexes, two intense bands corresponding to ligand-centered spin-allowed π–π* transitions appear at higher energy, with complex **8** exhibiting a red-shift of about 50 nm relative to complex **7**. The observed trend in the absorption profiles of complexes **7** and **8** parallels that of the free ligands (Figure S22).

**Table 4:** Photophysical Data of Complexes **7** and **8** and Proligands Hpbpz and Hpbpn in Acetonitrile Solution

		λ_PL_ (nm)					
Comp.	λ, nm (ε × 10^–4^ M^–1^·cm^–1^)^a^	RT^b^	77 K^c^	φ_PL_ ^b^	τ^b^ (μs)	*k*_r_ ^d^ (s^–1^ × 10^–4^)	*k*_nr_ ^d^ (s^–1^ × 10^–6^)
**7**	273 (6.37), 290 (2.84), 302 (2.47), 351 (0.94), 386 (0.92), 411 (0.81)	702	546, 592, 646, 706	0.004	0.71	0.6	1.4
**8**	300 (8.44), 333 (3.94), 398 (0.99), 420 (1.23), 448 (1.04)	564	504, 540, 576, 624	0.012	0.22	5.6	4.6
**Hpbpz**	308 (1.84), 350 (1.21), 366 (1.98), 388 (2.27)	508	n.m.	n.m.	n.m.	n.m.	n.m.
**Hpbpn**	302 (3.35), 376 (0.48), 396 (1.06), 420 (1.45)	544	n.m.	n.m.	n.m.	n.m.	n.m.

a Measured at room temperature in degassed acetonitrile solution at 1.0 × 10^–5^ M.

b Measured at room temperature in degassed acetonitrile solution at 1.0 × 10^–3^ M for **7** and 1.0 × 10^–5^ M for **8**, Hpbpz and Hpbpn.

c Measured in a 1.0 × 10^–4^ M glassy solution of EtOH:MeOH:CH_2_Cl_2_ (8:2:1).

d Radiative decay rate *k*_r_ = ϕ/τ and nonradiative decay rate *k*_nr_ = (1 – ϕ)/τ. n.m. = not measured.

**Table 5:** Photophysical Data of Complexes **7** and **8** in Solid State

		λ_PL_(nm)		τ (μs)				
Complex	λ (nm)^a^	RT	77 K	RT	77 K	φ_PL_ ^b^	*k*_r_ ^b^,^c^ (s^–1^ × 10^–4^)	*k*_nr_ ^b^,^c^ (s^–1^ × 10^–6^)
**7**	276, 317, 414	760	748	0.52	2.67	0.049	9.4	1.8
**8**	297, 315, 443, 470	856	844	0.81	0.60	0.012	1.4	1.2

a Diffuse reflectance solid state measurements.

b Measured at room temperature.

c Radiative decay rate *k*_r_ = ϕ/τ and nonradiative decay rate *k*_nr_ = (1 – ϕ)/τ.

In the solid state, both complexes exhibit absorptions at longer wavelengths, with absorption tails extending beyond 500 nm for **7** and 550 nm for **8** (Figure 6b). The red shift observed when comparing the absorption profiles in solution and in the solid state (Figure S23) appears to be associated with intermolecular π–π interactions present in the solid state.

**Figure 6:**
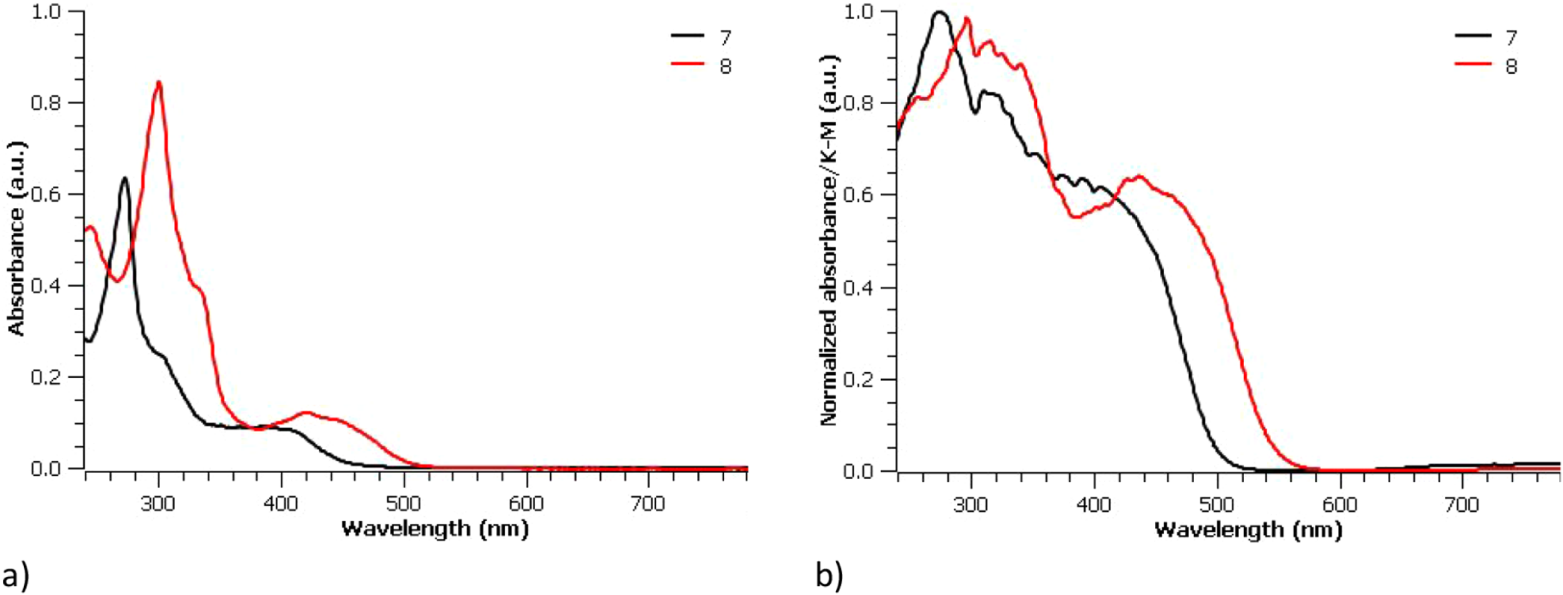
UV-vis absorption spectra of complexes **7** and **8** in degassed acetonitrile solution at 1.0 × 10^–5^ M (a) and normalized UV-vis absorption spectra in solid state (b) at room temperature.

Complexes **7** and **8** show solid-state photoluminescence at room temperature and at 77 K. The excitation spectra associated with the NIR emission do not reproduce the low-energy absorption tails in the solid state, indicating that population of the emissive triplet state is not dominated by the most intense allowed absorptions but proceeds via efficient ISC promoted by the heavy-atom effect. The microsecond lifetimes corroborate phosphorescence, without implying direct triplet excitation (Figures 6b, S24 and S25). The lifetime measurements are in the microsecond range (see Table 5 and Figures S27–S30). The emission spectra show low-energy maxima near or in the near-infrared region. Among them, complex **8**, with the highest conjugation shows a red-shifted emission if compared to complex **7**, following the same trend as in the absorption spectra, suggesting the participation of quinoxaline ligands in the orbitals responsible for the emissive behavior (see calculations section). The quantum yields of emission at room temperature are 4.9% and 1.2% for **7** and **8**, respectively. Thus, complexes **7** and **8** are solid state NIR emitters. Both emissions are blue-shifted at 77 K. This behavior, although rare, can be rationalized by considering the solid-state ambient rigidity.^33^ This curious atypical dependence on the temperature, which is termed as luminescence rigidochromism in other luminescent systems, has its origin in an effect that relates the emission features with the environmental rigidity in charge transfer states formed in transitions from a fluid to a rigid solvent. Different to that in solution, in the solid state this phenomenon is not fully understood, since in transitions at low temperatures one would expect a shortening of the metal–ligand or intraligand distances, which would lead to a reduction of the HOMO–LUMO band gap and, consequently, a red shift of the emission energy. In this case, this rigidochromism could be related to the short distances involving the metals and the ligands where further contraction provoked by decreasing temperature is unlikely.

The photophysical behavior in solution markedly differs from that observed in the solid state and highlights significant distinctions between the two complexes (Figure 7 and Figure S31–S33). For complex **7**, the emission spectra in acetonitrile and acetone solutions exhibit a pronounced concentration dependence: dilute solutions (10^–5^ M) are nonemissive, whereas increasing concentration induces luminescence accompanied by a progressive red shift (670 nm at 10^–4^ M; 702 nm at 10^–3^ M in acetonitrile; see Table 6 for the data of acetone solutions). In acetone, φ_PL_ measurements across various concentrations reveal a clear enhancement, consistent with an aggregation-induced emission (AIE) phenomenon. Given that the quantum yield represents the ratio of emissive species to the total population of excited molecules, its increase with concentration implies a change in the nature of the emissive species since otherwise, the ratio should be the same. This fact, together with the observed red shift, suggests an oligomerization process occurring in acetone solution at higher concentrations, potentially analogous to the aggregation pattern identified by X-ray diffraction analysis (Figure 3). In contrast, complex **8**, which features the longer π–conjugation exhibits emissive behavior that is independent of concentration and shows higher energy emission at 562 nm in acetonitrile (560 nm in acetone) in the 10^–5^–10^–3^ M range. A plausible rationale for the absence of a concentration effect on the emission of complex **8** is discussed in the computational analysis section (see below). For both complexes, the emissions observed in solution are more energetic than those in the solid state, indicating either a reduced strength or a diminished number of π–π interactions in solution compared to the solid state.

**Figure 7:**
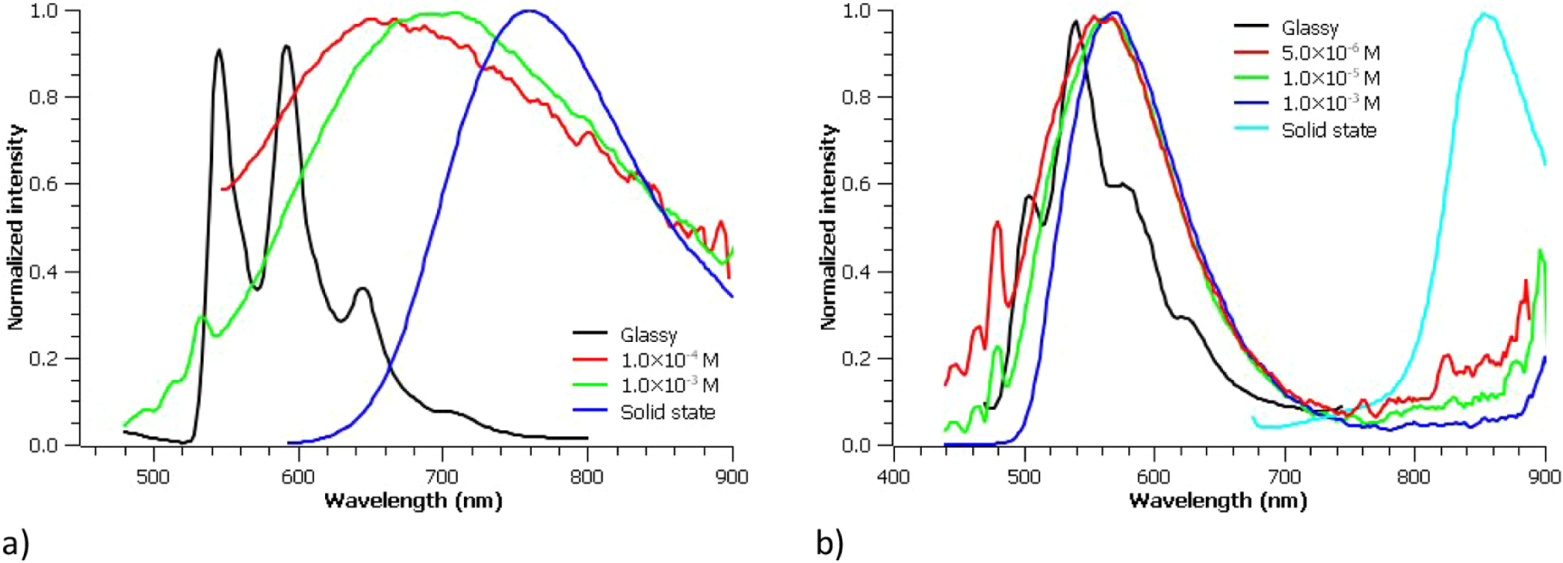
Normalized emission spectra of **7** (a) and **8** (b) at different concentrations in acetonitrile solution, in glassy solution and in solid state.

**Table 6:** Data of Emission of Complex **7** at Different Concentrations in Acetone and in the Solid State

Concent. (M)	λ_em_ (nm)	φ_PL_
2.5 × 10^–4^	660	0.005
2.5 × 10^–3^	666	0.034
2.5 × 10^–2^	690	0.051
Solid state	760	0.052

The lifetimes of both complexes in solution, as it was found in the solid state, fall within the microsecond range (Figure S26), with complex **8** exhibiting a shorter value. This behavior is consistent with phosphorescent emission for both complexes.

The emission spectra of glassy solutions (EtOH:MeOH:CH_2_Cl_2_, 8:2:1) show in both cases vibrationally resolved (1157–1423 cm^–1^) high energy emissions centered at 600 nm (**7**) and 550 nm (**8**) (see Figure 7), indicating a high contribution of the quinoxaline ligands in the emissive state in both complexes. To confirm the nature of these vibronic spacings, the IR spectra of both complexes and of the proligands Hpbpz and Hpbpn were recorded (Figures S34–S35). In all cases, several IR bands appear in the aforementioned region. The observed vibronic structure is typical of the vibrational modes of C^N ligands, as can be determined by comparison of the IR spectra, indicating that both the pbpz and pbpn ligands participate in the **7** and **8** emitting states, respectively.^34–38^

It is noteworthy that the emission spectrum of the glassy solution of complex **8** closely resembles those recorded in acetonitrile and acetone solutions, whereas the spectrum of complex **7** is blue-shifted relative to its solution-phase spectra. This finding further supports the interpretation of a progressive red shift with increasing concentration, indicative of a higher degree of aggregation in complex **7**—a feature not observed for complex **8**.

It is important to emphasize that, to the best of our knowledge, complexes **7** and **8** represent the first half-sandwich metal compounds reported to exhibit NIR emission. Furthermore, complexes **7** and **8** are NIR emitters in the solid state, with complex **7** displaying aggregation-enhanced emission (AEE). In the case of iridium derivatives, studies in this field have predominantly focused on bis­(cyclometalated) octahedral complexes. For this class of compounds, phosphorescence quantum yields as high as approximately 30% have been achieved in thin films; however, typical φ_PL_ values are considerably lower, and the emission wavelengths often remain close to 700 nm.^7,39–42^ Light-emitting electrochemical cells (LECs) constructed from such complexes have been reported with φ_PL_ values of 4.6%.^43^ Moreover, φ_PL_ values generally decrease significantly for emissions beyond 800 nm, as observed for complex **8**.

### Computational Studies

The interesting NIR luminescence displayed by complexes **7** and **8** in solid state and in solution (in the case of **7**) prompted us to perform an in-depth computational study (see Computational Details). We carried out DFT calculations on model systems of complexes **7** and **8** represented by two molecular units, to show the π-stacking observed in concentration-dependent aggregation in CH_3_CN solutions and in the solid-state structures and compute their influence on the photophysical properties. Previous DFT studies on mononuclear units of these complexes explained their expected properties in low concentrated solutions, where isolated mononuclear units were expected.^1^ Following the photophysical results in the previous section, we have performed several computational tasks. First, we performed the full optimization of models **7a** and **8a** in the ground state (S_0_) (**7a** and **8a** are the dimers of **7** and **8**, respectively). The analysis of the most important intermolecular structural parameters agrees well with the experimental ones, confirming the presence of π-stacking interactions and additional C–H···π interactions between mononuclear units of both complexes, giving rise to head-to-tail arrangements.

To confirm the existence of π-stacking interactions between the aromatic rings of the pbpz in complex **7** and pbpn in complex **8**, we computed the dissociation energies in the ground state, obtaining very weak interactions of −3.9 (**7a**) and −5.3 (**8a**) kJ·mol^–1^ that held dimer systems in acetonitrile solution.

To decipher the main characteristics of the intermolecular interactions between fragments in complexes **7** and **8** in solution and in solid state, we used the Independent Gradient Model based of Hirshfeld partition of molecular density (IGMH) method. This analysis of the electron density consists of a real-space function based on the Reduced Density Gradient (RDG), that allows visualization of covalent and noncovalent interactions in 3D isosurfaces.^44^ The IGMH approach provides some additional features to those of NCI calculations,^45^ being the separation of inter- and intramolecular interactions in different isosurfaces, better defined isosurfaces and multiple quantitative indexes that allow straightforward comparisons between different molecular systems. The RDG shows high values in regions far from the molecule and near-zero values where interactions occur. Low gradient and low density indicate weak interactions, while low gradient and high density point to stronger ones. The sign of the second-largest eigenvalue (λ_2_) of the electron density Hessian is used to distinguish interaction types: positive λ_2_ indicates repulsion, negative indicates attraction. These features are visualized as isosurfaces of sign­(λ_2_)·ρ_e_(r), colored using a Blue-Green-Red (BGR) scale: blue-green for attractive, green for van der Waals, and green-red for repulsive interactions. The mapped IGMH isosurfaces for models **7a** and **8a** in the ground state are depicted in Figure 8.

**Figure 8:**
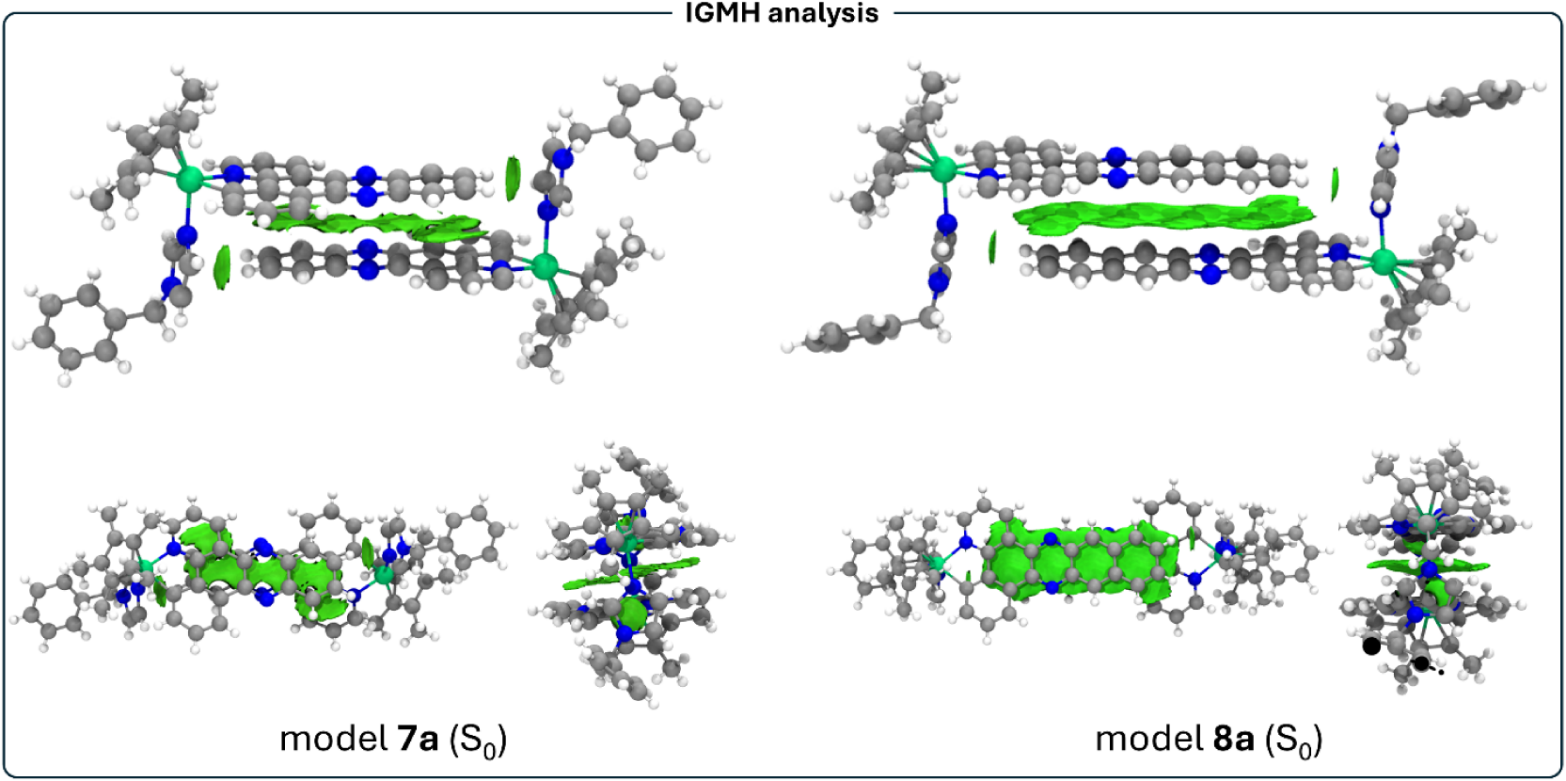
IGMH isosurfaces computed (isovalue = 0.005) for model systems **7a** and **8a** in the S_0_ ground state.

The analysis of the isosurfaces for both models shows up interesting features. The most important one is that π-stacking van-der Waals type interactions takes place between the quinoxaline (**7a**) and the benzoquinoxaline units (**8a**) between pairs of molecular complexes, as represented by a large green isosurface in each case. Additionally, for each model system two C–H···π interactions between C–H units of the extended quinoxaline systems and the π density of the imidazolyl units reinforce the possible existence of head-to-tail arrangements of dinuclear systems in acetonitrile solutions and confirm the observed solid-state dispositions.

On the other hand, the analysis of the frontier molecular orbitals for **7a** and **8a** shows that when we compare the HOMO–LUMO band gap of the dinuclear models with their corresponding mononuclear counterparts a clear reduction of the energy of the lowest virtual orbitals is obtained (Figure 9). This fact would be related to the presence of stabilizing π-stacking interactions and that it is manifested in the low energy red (**7**) or NIR (**8**) emissions found experimentally.

**Figure 9:**
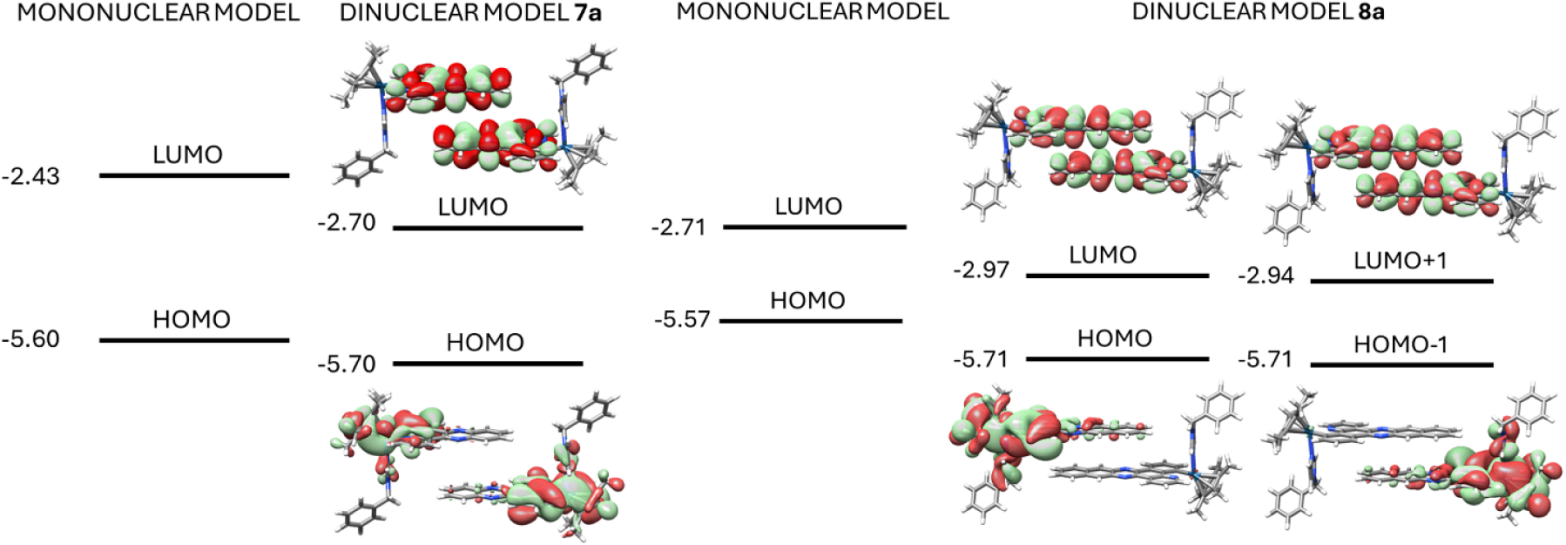
Frontier MO for model systems **7a** and **8a** in the S_0_ ground state.

The first 50 singlet–singlet excitations were computed for optimized model systems **7a** and **8a** at TD-DFT level of theory. The computed electronic excitations have been exported as spectrum profiles, and they are depicted in Figure 10. As it is observed, the computed spectra are very similar to the experimental ones shown in Figure 6a, showing high energy intense absorptions and a low energy absorption in each case. In addition, the computed results for dinuclear models also reflects the red-shift of the absorptions for model **8a**, in agreement with the previously commented stronger electron-withdrawing nature of the pbpn ligand due to a greater π-conjugation, as it was discussed previously.^1^

**Figure 10:**
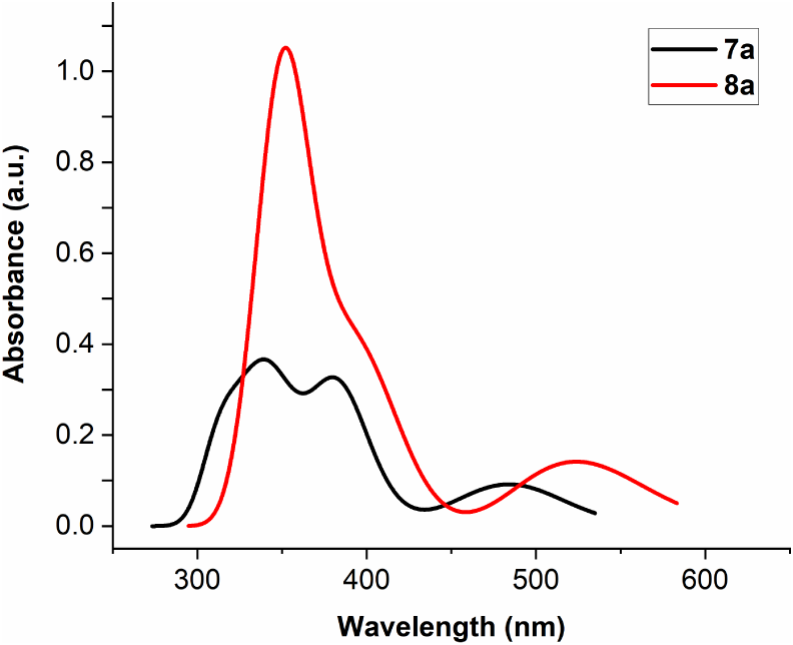
TD-DFT simulated UV-vis spectrum profiles for models of complexes **7a** and **8a**, by representing the first 50 singlet–singlet excitations.

We have also analyzed the character of the lowest electronic excitations, related to the experimentally observed emissive behavior of the reported complexes. Table 7 displays the nature of the computed S_0_→S_1_ and S_0_→T_1_ excitation energies for models **7a** and **8a**, which are also compared with the previously computed for mononuclear model systems.^1^

**Table 7:** Vertical Excitation Energies (eV) of S_1_ and T_1_ States (Values in Parentheses Correspond to Experimental Excitation Maxima) and Dominant Contributions to the Calculated Transitions for **7a** and **8a** Obtained at the TD-DFT (SMD, Acetonitrile)/6-31G­(d,p)//SDD Level

	TD-DFT Excitation^b^		Experimental Excitation^c^	Electronic transitions	
Complex	S_0_→S_1_	S_0_→T_1_	Solution/Solid	S_0_→S_1_	S_0_→T_1_
**7a (monomer of 7a)** ^a^	2.50 (2.66)	2.29 (2.31)	3.80/2.16	HOMO–1**→**LUMO	HOMO–6**→**LUMO
				HOMO**→**LUMO+1	HOMO–6**→**LUMO+1
**8a (monomer of 8a)** ^a^	2.28 (2.39)	1.49 (1.51)	2.95/1.89	HOMO**→**LUMO	HOMO–4**→**LUMO
				HOMO**→**LUMO+1	HOMO–4**→**LUMO+1

a Data from ref 1.

b Data computed in CH_3_CN as explicit solvent.

c Experimental excitation maxima in 10^–4^ M CH_3_CN solution/solid state.

The computed S_0_→S_1_ vertical excitations appear slightly red-shifted with respect to the low energy part of the experimental absorption spectrum and with respect to the previously computed monomer model systems. The computed S_0_→T_1_ vertical excitation, responsible for the phosphorescent behavior of complexes **7** and **8**, appears for model **7a** (2.29 eV) between those of solution (3.80 eV) and solid state (2.16 eV), and in the case of model **8a** (1.49 eV), appears below that of solution (2.95 eV) and close to that of solid state (1.89 eV), pointing to the importance of π-stacking interactions in the emissive properties of the complexes in solid state.

If we focus on the emissive character of compounds **7** and **8**, we can use **7a** and **8a** as models for a qualitative explanation of their optical properties. Thus, the MOs involved in the S_0_→T_1_ vertical excitation for **7a** and **8a** are depicted in Figure 11. As it can be observed, the origin of the phosphorescent properties of these complexes in solid state and in solution could be ascribed to an admixture of a ^3^LC (π→π*) transition with some contribution from a ^3^MLCT (d→π*) in the case of model **7a**, whereas a pure ^3^LC (π→π*) transition would be responsible for the phosphorescent properties of model **8a**. The possibly pure ^3^LC character of the emission of complex **8a** is further corroborated by the similarity between the emission spectra of the complex and the Hpbpn ligand (Figure S36 and S37).

**Figure 11:**
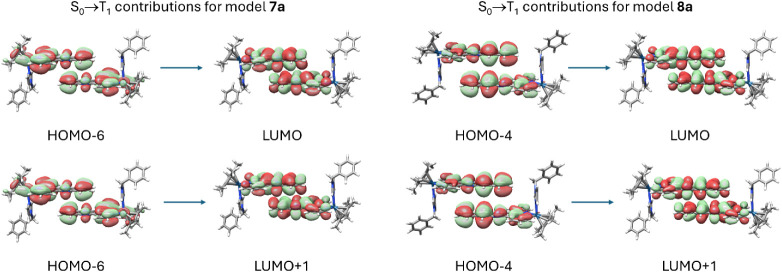
Frontier MOs involved in the lowest S_0_→T_1_ vertical excitations for **7a** and **8a**.

We also computed the optimization of models **7a** and **8a** in the lowest triplet excited state (T_1_) from which the phosphorescent emissions take place. In both cases, the optimized structures still display π-stacking interactions but at very large distances and with less eclipsed aromatic systems, in agreement with the population of π* orbitals of the quinoxaline and benzoquinoxaline units. We have computed the IGMH isosurfaces that show a very weak van der Waals interaction between quinoxaline (**7a**) and benzoquinoxaline (**8a**) moieties (Figure 12). One of the C–H···π interactions between units is lost in the dimers **7a** and **8a**. The fact that these interactions appear very weak in the lowest triplet excited state upon irradiation could be directly related to the observed behaviors. This distortion of the triplet excited states is higher for **8a** than for **7a** (Figure 12). Although the calculated model represents a very simplified state of the oligomerization in both complexes, we propose that the greater structural distortion observed for **8a** compared to **7a** may account for the distinct influence of solution concentration on the emission behavior of the two complexes. That is to say that the distinct emission behavior of complexes **7** and **8** found experimentally could be attributed to differences in their excited-state distortion. Thus, complex **8**, with its more extended π-system, likely undergoes a greater structural rearrangement upon excitation, which disrupts π–π stacking and favors emission from monomeric species in solution. This phenomenon is reminiscent of cases in which twisted excited state molecular conformations have been reported.^46^ In contrast, complex **7** retains a geometry closer to its ground state, allowing aggregates to persist in the excited state and enabling aggregation-enhanced emission. In the solid state, however, the molecular proximity favors emission from aggregated species. Thus, in the case of complex **7**, the observed tendency to oligomerization (emission red-shift with increasing concentrations), with less distorted triplet excited states and closer π-stacking interactions, favors the emitting ^3^LC (π→π*)/^3^MLCT (d→π*) emissive origin. The energy difference between solution and solid-state emission for **8** may reflect the energetic cost of breaking π–π interactions during disaggregation.

**Figure 12:**
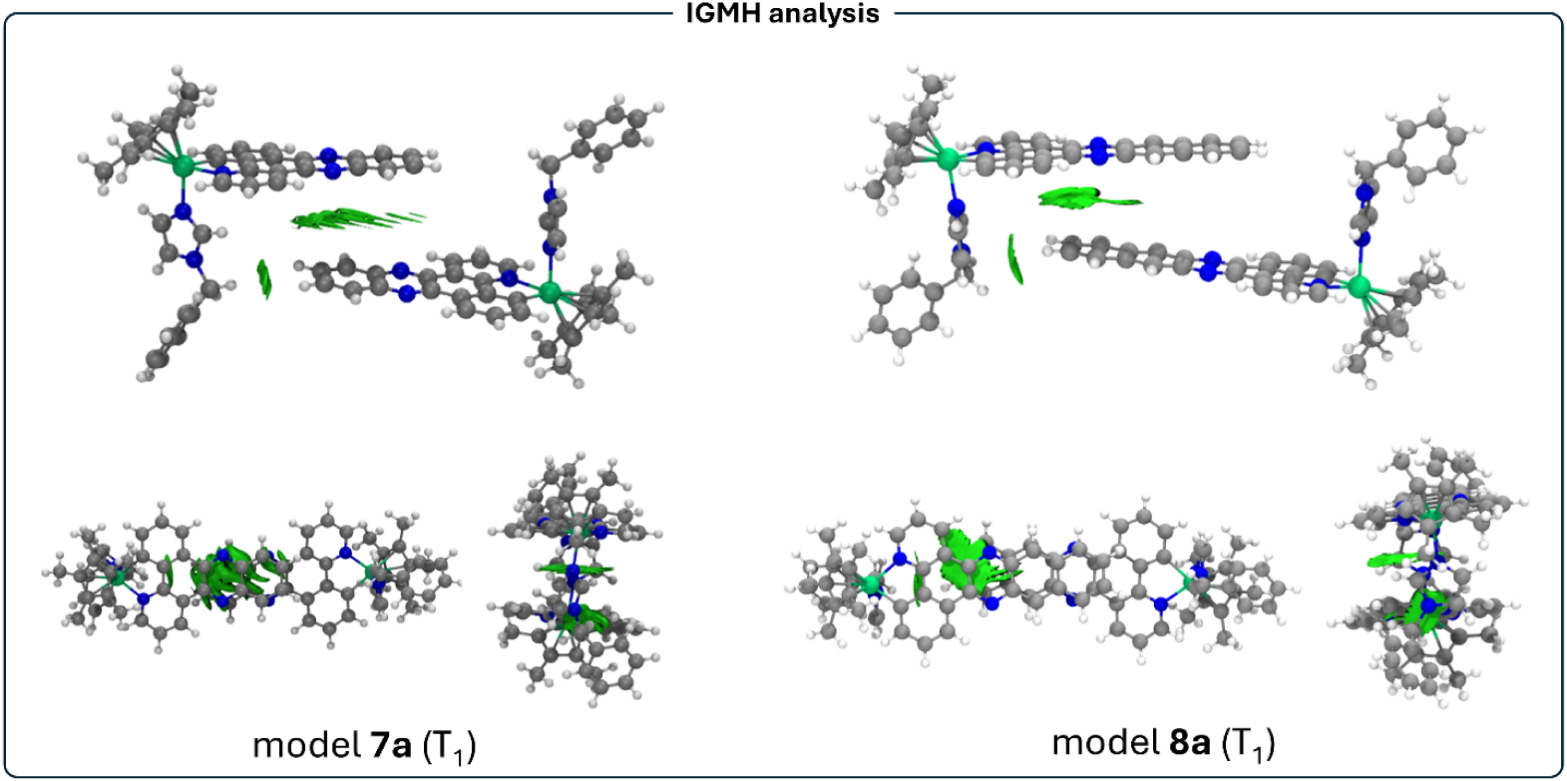
IGMH isosurfaces computed (isovalue = 0.0005) for model systems **7a** and **8a** in the T_1_ excited state.

## Conclusions

We have studied the aggregation and their influence on the photophysical properties of half-sandwich cyclometalated iridium complexes of the type [Cp*Ir­(C^N)*L*]­BF_4_ with the C^N π-extended ligands pbpz (4,9,14-triazadibenzo­[*a*,*c*]­anthracene) or pbpn (4,9,16-triazadibenzo­[*a*,*c*]­naphthacene). The pbpn complexes contain one extra fused ring. Aggregation was observed by ^1^H NMR and PFGSE-DOSY NMR experiments at different concentrations, with a more intense effect for the pbpn complexes. X-ray diffraction revealed that head-to-tail dimers were formed by π–π and CH−π interactions with pair of enantiomers. The π–π interactions in the dimers were stronger for the pbpn complexes. In the case of some complexes with the pbpz ligand the dimers were additionally aggregated through π–π interactions forming infinite polymeric chains while in a similar complex with pbpn, formation of π–π interactions between dimers were not observed giving rise to a more open structure.

The complexes with *L* = *N*-benzylimidazole (C^N = pbpz, **7**; pbpn, **8**) were studied more in depth. By DLS (dynamic light scattering) in aqueous solutions it was verified that both complexes formed aggregates, which were stable over time, with complex **8** aggregating at lower concentration, whereas complex **7** produced larger aggregates.

When studying the photophysical properties of **7** and **8**, a notable effect of the extra ring was found. Both complexes become NIR emitters in solid state with a red-shift for **8**, the complex with the more π-expansive ligand. They are the first half-sandwich metal complexes exhibiting NIR emission activity. Both complexes display phosphorescence in the solid state and in solution. In acetonitrile or acetone solutions, a different effect of increasing concentration was found in the two complexes. In the case of complex **7**, a red-shift and an increase in PLQY were observed with increasing concentration, indicating aggregation-enhanced emission (AEE). In contrast, complex **8** shows no shift in emission wavelength with increasing concentration; however, a pronounced red-shift is observed upon transitioning from solution to the solid state.

DFT and TD-DFT studies support the experimentally observed features through the study of dinuclear model systems (**7a** and **8a)** displaying π–π stacking interactions, which are of paramount importance for the description of their NIR-emissive behavior. A qualitative explanation of the phosphorescent properties has been achieved. Thanks to the TD-DFT results and the IGMH isosurfaces computed, it has been found that the triplet excited states undergo structural distortion, which is more pronounced in **8a** than in **7a**. Although the calculated model represents a very simplified state of the oligomerization, we propose that the greater structural distortion observed for **8a** may account for the distinct influence of solution concentration on the emission behavior of the two complexes. In complex **7**, the lower distortion would enable oligomerization to persist in the excited state, whereas the pronounced distortion in **8** would likely disrupt aggregation, resulting in emission predominantly from low-nuclearity species. In the solid state, however, the molecular proximity favors emission from aggregated species.

In conclusion, thanks to the introduction of the π-expansive ligands, and the subsequent aggregation processes observed, NIR-emitting complexes in the solid state have been obtained and also a complex with aggregation-enhanced emission (AEE). Furthermore, a very notable effect on the aggregation and photophysical properties of the presence or not of the extra ring in the C^N ligands has been evidenced. The discovery of the first half-sandwich complexes with NIR emission may open new avenues for future significant developments.

## Experimental Section

### Photophysical Properties

Excitation and emission spectra in the solid state (RT and 77 K) and in degassed acetonitrile and acetone solutions were recorded using an Edinburgh FLS1000 fluorescence spectrometer. Luminescence lifetime was measured on an Edinburgh FLS1000 fluorescence spectrometer. Excitation and emission spectra of glassy solutions (EtOH:MeOH:CH_2_Cl_2_, 8:2:1) were recorded on a Shimadzu RF-6000 fluorescence spectrometer equipped with a quartz dewar for measurements at 77 K. The quantum yields were measured in the solid state using a Hamamatsu Quantaurus-QY C11347-11 integrating sphere.

### Analysis of π–π Stacking by ^1^H NMR Spectroscopy

Solutions of selected complexes were prepared at different concentrations ranging from 0.025 to 25 mM in CDCl_3_ or acetone-*d*_6_ and analyzed by ^1^H NMR spectroscopy.

### ^1^H NMR and PFGSE NMR Concentration-Dependent Experiments

These studies (2 mM, 12 mM, 25 mM) were carried out using a Bruker Avance Neo NMR spectrometer operating at 500.16 MHz for ^1^H. The spectrometer was equipped with an iProbe NMR probe (Bruker, Germany). High-quality 5 mm NMR tubes (Wilmad LabGlass) were used to contain the solutions (500 μL). TMS was added to each sample in an amount corresponding to 1.3 equiv. relative to compound **7** or **8**, ensuring a consistent ratio between the compound of interest and TMS across all samples. The rotation of the sample and the temperature control were switched off before running the PFGSE NMR experiments to minimize thermal convection currents. The pulse width (*p*1) was optimized for every sample before the PFGSE experiment. The ledbpgp2s pulse sequence from the Bruker library was chosen to run the PFGSE NMR experiments, with 16 increments in the gradient strength (5–95%), typically 16 averages per increment step, and 75 ms as the diffusion time. The diffusion coefficients, *D*_t_, were calculated using the standard Bruker software (Table 2, Table S6)

### Dynamic Light Scattering (DLS)

The aggregation of the complexes in aqueous solution was evaluated by DLS. Solutions of complexes **7** and **8** at concentrations ranging from 25 to 250 μM in aqueous solution (10% DMSO) were studied and the hydrodynamic diameter of the particles at 25 °C were measured using a Dynamic Light Scattering (DLS, ZetaPlus, Brookhaven, Holtsville, NY) instrument operating at a 90-scattering angle with a 635 nm (35 mW) diode laser source. The path length of the cuvette was 1 cm. The stability of the aggregates was monitored after 4 hours.

### X-ray Crystallographic Structure Determination

Suitable single crystals for the X-ray diffraction study were obtained in the following way. **3**: by slow diffusion of pentane into a solution of **3** in DCM. **7**: by slow diffusion of pentane into a solution of **7** in acetone. The complexes crystallize in the space group *P*2_1_/*n* of the monoclinic system (**3**) or in *PI̅* of the triclinic system (**7**).

Data collection and refinement parameters for complexes **3** and **7 × 0.75C**_**2**_ **H**_**6**_ **O** are summarized in Table S1. Suitable single crystals of compounds **3** and **7** were mounted on a Bruker APEX II and Bruker Venture CCD diffractometer with Diamond micro source MoKα (λ = 0.71073 Å) radiation. The data sets were integrated with Saint^47^ and corrected for Lorentzian and polarization effects. A semiempirical absorption correction was applied to the diffraction data.^48,49^ The software packages Wingx and OLEX2 ^50–52^ were used for space group determination, structure solution, and refinement by full-matrix least-squares methods based on *F* ^2^. A successful solution by direct methods provided most non-hydrogen atoms from the *E* map. The remaining non-hydrogen atoms were located in an alternating series of least-squares cycles and difference Fourier maps. The non-hydrogen atoms were refined with anisotropic displacement coefficients and hydrogen atoms were placed by using a riding model and included in the refinement at calculated positions.

Exceptions and special features: for compound **7 × 0.75C**_**2**_ **H**_**6**_ **O**, several plate and block crystals were chosen, and all were found to be twinned. The selected crystals were indexed as a two-component nonmerohedral twin related by a rotation of 180°.^53^ The reflections from the two domains were simultaneously integrated and Twinabs was used for scaling, empirical absorption corrections and the generation of two different data files, one with detwinned data for structure solution and a second one for structure refinement against total integrated intensities. For compound **3**, the unit cell shows two voids of about 133 Å^3^ each, probably filled with some highly disordered pentane solvent that could not be satisfactorily refined. The Squeeze procedure^54^ was therefore used to remove mathematically the effect of the solvent. The quoted formula and derived parameters do not include the squeezed solvent molecules. The tetrafluoroborate counteranion was found disordered over two positions and several restraints (DELU, SIMU and DFIX) were used in order to improve refinement stability.

Deposition numbers in the Cambridge database are 2223320 for **3** and 2223322 for **7 × 0.75C**_**2**_ **H**_**6**_ **O**.

### Computational Details

Model systems **7a** and **8a** were fully optimized in the ground (S_0_) and lowest triplet (T_1_) excited states using the Gaussian 16 Revision C.01 suite of programs^55^ at the DFT level of theory using the B3LYP functional.^56,57^ Dispersion corrections were introduced within the D3-Grimme correction,^58^ whereas solvent effects were considered within the self-consistent reaction field (SCRF) theory using the solvation model SMD.^59^ The 6-31G­(d,p)^60^ basis set was used for C, N, and H atoms and the SDD effective core potential basis set for Ir atom.^61,62^ The structures and molecular orbitals were visualized and rendered using GaussView 6.1 ^63^ and UCSF ChimeraX 1.3 visualization programs.^64^ TD-DFT calculations were performed to compute the absorption spectra for models **7a** and **8a** and the character of the MOs involved in the lowest singlet–singlet and singlet–triplet excitations. A topological analysis on model systems **7a** and **8a** was performed with the aim of analyzing the interaction nature between mononuclear fragments in real space and from a qualitative point of view. The topology and properties of the DFT electron density of the structures was analyzed using independent gradient model based on Hirshfeld partition (IGMH)^44^ methods using Multiwfn 3.8 software.^65,66^ Additionally, VMD 1.9.4a^67^ visualization program package was employed for the representations of the electron density studied in each analysis.

## Supplementary Material



## Notes

Data from X-ray diffraction, NMR spectroscopy, DLS experiments, photophysical properties, and IR spectra; CCDC 2223320 and 2223322 contain the supplementary crystallographic data for this paper (PDF)

## References

[1] Gonzalo-Navarro C., Zafon E., Organero J. A., Jalón F. A., Lima J. C., Espino G., Rodríguez A. M., Santos L., Moro A. J., Barrabés S., Castro J., Camacho-Aguayo J., Massaguer A., Manzano B. R., Durá G. (2024). Ir­(III) Half-Sandwich Photosensitizers
with a π-Expansive Ligand for Efficient Anticancer Photodynamic
Therapy. J. Med. Chem..

[2] Kim D. Y., Song D. W., Chopra N., De Somer P., So F. (2010). Organic Infrared
Upconversion Device. Adv. Mater..

[3] Lee E. C., Jun H., Kim D. (2011). New Finger Biometric
Method Using Near Infrared Imaging. Sensors.

[4] Kim H. U., Kim T., Kim C., Kim M., Park T. (2023). Recent Advances in
Structural Design of Efficient Near-Infrared Light-Emitting Organic
Small Molecules. Adv. Funct. Mater..

[5] Lee W. W. H., Zhao Z., Cai Y., Xu Z., Yu Y., Xiong Y., Kwok R. T. K., Chen Y., Leung N. L. C., Ma D. (2018). Facile access to deep red/near-infrared emissive
AIEgens for efficient non-doped OLEDs. Chem.
Sci..

[6] Shafikov M. Z., Pander P., Zaytsev A. V., Daniels R., Martinscroft R., Dias F. B., Williams J. A. G., Kozhevnikov V. N. (2021). Extended
ligand conjugation and dinuclearity as a route to efficient platinum-based
near-infrared (NIR) triplet emitters and solution-processed NIR-OLEDs. J. Mater. Chem. C.

[7] Pashaei B., Karimi S., Shahroosvand H., Pilkington M. (2020). Molecularly
Engineered Near-Infrared Light-Emitting Electrochemical Cells. Adv. Funct. Mater..

[8] Costa R. D., Ortí E., Bolink H. J. (2011). Recent advances in light-emitting
electrochemical cells. Pure Appl. Chem..

[9] Chelushkin P. S., Shakirova J. R., Kritchenkov I. S., Baigildin V. A., Tunik S. P. (2022). Phosphorescent NIR
emitters for biomedicine: applications,
advances and challenges. Dalton Trans..

[10] Liu Y., Zhang P., Fang X., Wu G., Chen S., Zhang Z., Chao H., Tan W., Xu L. (2017). Near-infrared
emitting iridium­(III) complexes for mitochondrial imaging in living
cells. Dalton Trans..

[11] Shen Q., Gao K., Zhao Z., Gao A., Xu Y., Wang H., Meng L., Zhang M., Dang D. (2023). Aggregation-induced
emission (AIE)-active metallacycles with near-infrared emission for
photodynamic therapy. Chem. Commun..

[12] Chen G.-Y., Chang B.-R., Shih T.-A., Lin C.-H., Lo C.-L., Chen Y.-Z., Liu Y.-X., Li Y.-R., Guo J.-T., Lu C.-W. (2019). Cationic
Ir^III^ Emitters with Near-Infrared
Emission Beyond 800 nm and Their Use in Light-Emitting Electrochemical
Cells. Chem. - Eur. J..

[13] Xue J., Xin L., Hou J., Duan L., Wang R., Wei Y., Qiao J. (2017). Homoleptic Facial Ir­(III) Complexes via Facile Synthesis for High-Efficiency
and Low-Roll-Off Near-Infrared Organic Light-Emitting Diodes over
750 nm. Chem. Mater..

[14] Gupta A. K., Kumar A., Singh R., Devi M., Dhir A., Pradeep C. P. (2018). Facile Synthesis
of an Organic Solid State Near-Infrared-Emitter
with Large Stokes Shift via Excited-State Intramolecular Proton Transfer. ACS Omega.

[15] Das B. (2025). Unveiling
mechanistic insights and applications of aggregation-enhanced emission
(AEE)-active polynuclear transition metal complexes. Chem. Commun..

[16] Liu Z., Habtemariam A., Pizarro A. M., Clarkson G. J., Sadler P. J. (2011). Organometallic
iridium­(III) cyclopentadienyl anticancer complexes containing C,N-Chelating
ligands. Organometallics.

[17] Millett A. J., Habtemariam A., Romero-Canelón I., Clarkson G. J., Sadler P. J. (2015). Contrasting
Anticancer Activity of Half-Sandwich Iridium­(III)
Complexes Bearing Functionally Diverse 2-Phenylpyridine Ligands. Organometallics.

[18] Wang C., Chen H. Y. T., Bacsa J., Catlow C. R. A., Xiao J. (2013). Synthesis
and X-ray structures of cyclometalated iridium complexes including
the hydrides. Dalton Trans..

[19] Pracharova J., Vigueras G., Novohradsky V., Cutillas N., Janiak C., Kostrhunova H., Kasparkova J., Ruiz J., Brabec V. (2018). Exploring
the Effect of Polypyridyl Ligands on the Anticancer Activity of Phosphorescent
Iridium­(III) Complexes: From Proteosynthesis Inhibitors to Photodynamic
Therapy Agents. Chem. - Eur. J..

[20] Yam V. W.-W., Lo K. K.-W., Cheung K.-K., Kong R. Y.-C. (1997). Deoxyribonucleic
acid binding and photocleavage studies of rhenium­(I) dipyridophenazine
complexes. J. Chem. Soc., Dalton Trans..

[21] Herebian D., Sheldrick W. S. (2002). Synthesis and DNA binding properties of bioorganometallic
(η^5^-pentamethylcyclopentadienyl)­iridium­(III) complexes
of the type [(η^5^-C_5_Me_5_)­Ir­(Aa)­(dppz)]^n+^ (dppz = dipyrido­[3,2-a: 2′,3′-c]-phenazine,
n = 1–3), with S-coordinated amino acids (Aa) or peptides. Dalton Trans..

[22] Lo K. K. W., Chung C. K., Zhu N. (2006). Nucleic acid
intercalators and avidin
probes derived from luminescent cyclometalated iridium­(III)-dipyridoquinoxaline
and -dipyridophenazine complexes. Chem. - Eur.
J..

[23] Manzano B. R., Jalón F. A., Carrión M. C., Durá G. (2016). Bis­(pyrazol-1-yl)­(pyridin-x-yl)­methane
Ligands – Mono- or Ditopic Ligands in Complexes and Supramolecular
Frameworks. Eur. J. Inorg. Chem..

[24] Reger D. L., Gardinier J. R., Semeniuc R. F., Smith M. D. (2003). Silver complexes
of 1,1′,3,3′-tetrakis­(pyrazol-l-yl)­propane: The ‘quadruple
pyrazolyl embrace’ as a supramolecular synthon. Dalton Trans..

[25] Reger D. L., Semeniuc R. F., Elgin J. D., Rassolov V., Smith M. D. (2006). 1,8-Naphthalimide
Synthon in Silver Coordination Chemistry: Control of Supramolecular
Arrangement. Cryst. Growth Des..

[26] Gómez-García C. J., Escrivà E., Mínguez Espallargas G., Borrás-Almenar J. J., Soto L., Sancho A., García-Lozano J., Ramírez De Arellano C. (2014). A rare example of nickel­(II) chains
based on a heteroscorpionate-like ligand with quadruple imidazolyl
interactions. Dalton Trans..

[27] Escrivà E., Folgado J. V., Sancho A., Ortíz R., Perelló L., de Arellano C. R. (2019). Cooperative
H-bonds, π···π
and anion···π interactions as driving forces
in the construction of novel Cu­(II) bis­(imidazol-2-yl) supramolecular
3D frameworks. Polyhedron.

[28] Dance I., Scudder M. (2009). Molecules embracing
in crystals. CrystEngComm.

[29] Carrión M. C., Durá G., Jalón F. A., Manzano B. R., Rodríguez A. M. (2012). Polynuclear
complexes containing ditopic bispyrazolylmethane ligands. Influence
of metal geometry and supramolecular interactions. Cryst. Growth Des..

[30] Xu Y., Wang X., Song K., Du J., Liu J., Miao Y., Li Y. (2021). BSA-encapsulated cyclometalated iridium
complexes as nano-photosensitizers for photodynamic therapy of tumor
cells. RSC Adv..

[31] Fujita D., Ueda Y., Sato S., Mizuno N., Kumasaka T., Fujita M. (2016). Self-assembly of tetravalent
Goldberg polyhedra from
144 small components. Nature.

[32] Durand E., Clemancey M., Lancelin J., Verstraete J., Espinat D., Quoineaud A. (2009). Aggregation
States of Asphaltenes:
Evidence of Two Chemical Behaviors by ^1^H Diffusion-Ordered
Spectroscopy Nuclear Magnetic Resonance. J.
Phys- Chem. C.

[33] Fernández E. J., Laguna A., López-de-Luzuriaga J. M., Monge M., Montiel M., Olmos M. E., Rodríguez-Castillo M. (2009). Unsupported
Au (I). Cu­(I) interactions: influence of nitrile ligands and aurophilicity
on the structure and luminescence. Dalton Trans..

[34] Berenguer J. R., Pichel J. G., Giménez N., Lalinde E., Moreno M. T., Piñeiro-Hermida S. (2015). Luminescent pentafluorophenyl-cycloplatinated
complexes: Synthesis, characterization, photophysics, cytotoxicity
and cellular imaging. Dalton Trans..

[35] Diez A., Forniés J., García A., Lalinde E., Moreno M. T. (2005). Synthesis,
structural characterization, and photophysical properties of palladium
and platinum­(II) complexes containing 7,8-benzoquinolinate and various
phosphine ligands. Inorg. Chem..

[36] Berenguer J. R., Lalinde E., Moreno M. T., Sanchez S., Torroba J. (2012). Facile Metalation
of Hbzq by [cis-Pt­(C_6_F_5_)_2_(thf)_2_]: A Route to a Pentafluorophenyl Benzoquinolate Solvate Complex
That Easily Coordinates Terminal Alkynes. Spectroscopic And Optical
Properties. Inorg. Chem..

[37] Berenguer J. R., Lalinde E., Martin A., Moreno M. T., Ruiz S., Sanchez S., Shahsavari H. R. (2014). Photophysical Responses in Pt_2_Pb Clusters Driven by Solvent Interactions and Structural
Changes in the Pb^II^ Environment. Inorg. Chem..

[38] Lalinde E., Lara R., López I. P., Moreno M. T., Alfaro-Arnedo E., Pichel J. G., Piñeiro-Hermida S. (2018). Benzothiazole-Based
Cycloplatinated Chromophores: Synthetic, Optical, and Biological Studies. Chem. - Eur. J..

[39] Zhang Y., Qiao J. (2021). Near-infrared emitting iridium complexes: Molecular design, photophysical
properties, and related applications. IScience.

[40] Li S., Zhang Y., Dai Y., Xu J., Meng Q.-Y., Wen X.-L., Qiao J. (2025). High-Efficiency Near-Infrared
Iridium
(III) Complexes with Tailored Ligands for Solution-Processed OLEDs
with Maximum EQE of 3.75% at 830 nm. Adv. Opt.
Mater..

[41] Zhu C., Liu L., Yang X., Zhou G., Sun Y. (2024). The Molecular
Design
and Electroluminescent Performance of Near-Infrared (NIR) Iridium
(III) Complexes Bearing Isoquinoline-, Phthalazine- and Phenazine-Based
Ligands. ChemPhysChem.

[42] Ni G., Yan J., Wu Y., Zhou F., Chou P. T., Chi Y. (2023). Transition-metal
phosphors with emission peak maximum on and beyond the visible spectral
boundaries. Inorg. Chem. Front..

[43] Constable E. C., Housecroft C. E., Schneider G. E., Zampese J. A., Bolink H. J., Pertegas A., Roldan-Carmona C. (2014). Red
emitting [Ir­(C^N)_2_(N^N)]^+^ complexes employing
bidentate 2,2’: 6’,2’’-terpyridine
ligands for light-emitting electrochemical cells. Dalton Trans..

[44] Lu T., Chen Q. (2022). Independent gradient
model based on Hirshfeld partition: A new method
for visual study of interactions in chemical systems. J. Comput. Chem..

[45] Johnson E. R., Keinan S., Mori-Sánchez P., Contreras-García J., Cohen A. J., Yang W. (2010). Revealing Noncovalent Interactions. J. Am. Chem. Soc..

[46] Gąsiorskia P., Matusiewicza M., Gondekb E., Uchaczc T., Daneld A., Wojtasikd K., Vlokhe R., Kityka A. V. (2017). Synthesis, UV–Vis
spectroscopy and DFT/TDDFT calculations on 6-substituted halogen derivatives
of 1,3-diphenyl-1 H-pyrazolo [3, 4 – b]­quinoxalines dyes. J. Lumin..

[47] SAINT v8.37, Bruker-AXS (2016) PEX3 v2016.1.0., Bruker AXS Inc.: Madison, Wisconsin, USA, 2016.

[48] Krause L., Herbst-Irmer R., Sheldrick G. M., Stalke D. (2015). Comparison of silver
and molybdenum microfocus X-ray sources for single-crystal structure
determination. J. Appl. Crystallogr..

[49] Sheldrick, G. M. ; Twinabs; University of Göttingen, Göttingen, Germany 2012.

[50] Farrugia L. J. (2012). WinGX and
ORTEP for Windows: An update. J. Appl. Crystallogr..

[51] Dolomanov O. V., Bourhis L. J., Gildea R. J., Howard J. A. K., Puschmann H. (2009). OLEX2: A complete
structure solution, refinement and analysis program. J. Appl. Crystallogr..

[52] Sheldrick, G. M. SHELX-2014, Program for Crystal Structure Refinement; University of Göttingen: Göttingen, Germany, 2014.

[53] Sheldrick, G. M. Cell_Now; University Of Göttingen: Göttingen, Germany, 2008.

[54] Van
Der Sluis P., Spek A. L. (1990). BYPASS: an effective method for the
refinement of crystal structures containing disordered solvent regions. Acta Crystallogr. Sect. A.

[55] Frisch, M. J. ; Trucks, G. W. ; Schlegel, H. B. ; Scuseria, G. E. ; Robb, M. A. ; Cheeseman, J. R. ; Scalmani, G. ; Barone, V. ; Petersson, G. A. ; Nakatsuji, H. Gaussian 16 Rev. C.01; Gaussian, Inc.: Wallingford CT, 2016.

[56] Becke A. D. (1992). Density-functional
thermochemistry. I. The effect of the exchange-only gradient correction. J. Chem. Phys..

[57] Lee C., Yang W., Parr R. G. (1988). Development of the Colle-Salvetti
correlation-energy formula into a functional of the electron density. Phys. Rev. B.

[58] Grimme S., Ehrlich S., Goerigk L. (2011). Effect of the damping function in
dispersion corrected density functional theory. J. Comput. Chem..

[59] Marenich A. V., Cramer C. J., Truhlar D. G. (2009). Universal solvation model based on
solute electron density and on a continuum model of the solvent defined
by the bulk dielectric constant and atomic surface tensions. J. Phys. Chem. B.

[60] Petersson G. A., Bennett A., Tensfeldt T. G., Al-Laham M. A., Shirley W. A., Mantzaris J. (1988). A complete basis set model chemistry. I. The total
energies of closed-shell atoms and hydrides of the first-row elements. J. Chem. Phys..

[61] Dunning, T. H., Jr. ; Hay, P. J. Modern Theoretical Chemistry; Plenum Press, 1977, Vol. 3; pp. 1–28.

[62] Andrae D., Häußermann U., Dolg M., Stoll H., Preuß H. (1990). Energy-adjusted
ab initio pseudopotentials for the
second and third row transition elements. Theor.
Chim. Acta.

[63] Dennington, R. ; Keith, T. A. ; Millam, J. M. GaussView, Version 6.1.1; Semichem, Inc.: Shawnee Mission, KS, 2019.

[64] Meng E. C., Goddard T. D., Pettersen E. F., Couch G. S., Pearson Z. J., Morris J. H., Ferrin T. E. (2023). UCSF ChimeraX:
Tools for structure
building and analysis. Protein Sci..

[65] Lu T., Chen F. (2012). Multiwfn: A multifunctional wavefunction analyzer. J. Comput. Chem..

[66] Lu T. (2024). A comprehensive
electron wavefunction analysis toolbox for chemists, Multiwfn. J. Chem. Phys..

[67] Humphrey W., Dalke A., Schulten K. (1996). VMD: Visual
Molecular Dynamics. J. Mol. Graph..

