# Supplementary material for: NIR-Emitting Cyclometalated Cp*-Ir(III) Complexes: Impact of Ligand π‑Extension on Aggregation Behavior and Photophysical Properties
Source: Inorg Chem. 2026 Jan 30;65(6):3477–90. doi: 10.1021/acs.inorgchem.5c05185 (PMC12914635; doi:10.1021/acs.inorgchem.5c05185)
Supplement: Supplementary file 1 [file ic5c05185_si_001.pdf]

Supporting Information

NIR-emitting cyclometallated Cp*-Ir(III) complexes: impact of ligand π-extension on aggregation behavior and photophysical properties

Carlos Gonzalo-Navarro,[1] María Rodríguez-Castillo,[2] Miguel Monge,[2] José M. López-de-Luzuriaga,[2] Félix A. Jalón,[1] Ana M. Rodríguez,[1,3] M. Victoria Gomez,[1] Gema Durá,[1,*] Blanca R. Manzano[1,*]

[1] Universidad de Castilla-La Mancha, Departamento de Química Inorgánica, Orgánica y Bioquímica- IRICA, Facultad de Ciencias y Tecnologías Químicas, Avda. C. J. Cela, 10, 13071 Ciudad Real, Spain.

[2] Departamento de Química, Instituto de Investigación en Química (IQUR), Universidad de La Rioja, Madre de Dios 53, 26006 Logroño, Spain.

[3] Escuela Técnica Superior de Ingenieros Industriales, Avda. C. J. Cela, 3, 13071 Ciudad Real, Spain.

Email address of the corresponding author: blanca.manzano@uclm.es

Index

Data from X-ray diffraction, p. S2-S7.

Data from NMR spectroscopy, p.S7-S14

DLS experiments, p.S15

Photophysical properties and IR spectra, p-S16-S21

X-ray diffraction

Table S1. Crystal data and structure refinement for **3** and **7 × 0.75C$_3$H$_6$O**.

	**3**	**7 × 0.75C$_3$H$_6$O**
Empirical formula	$C_{32}H_{29}BF_4IrN_5$	$C_{165}H_{158}B_4F_{16}Ir_4N_{20}O_3$
Formula weight	762.61	3585.14
Temperature (K)	293(2)	100(2)
Wavelength (Å)	0.71073	0.71073
Crystal system	Monoclinic	Triclinic
Space group	P 2_1/n	P $\bar{1}$
a (Å)	9.724(4)	14.0014(9)
b(Å)	23.182(9)	18.1827(12)
c(Å)	13.659(5)	30.125(2)
α(°)	90	86.035(2)
β(°)	93.706(5)	81.233(2)
γ(°)	90	89.462(2)
Volume(Å^3)	3073(2)	7561.7(9)
Z	4	2
Density (calculated) (g/cm^3)	1.649	1.575
Absorption coefficient (mm^{-1})	4.400	3.590
F(000)	1496	3568
Crystal size (mm^3)	0.19 x 0.09 x 0.08	0.90 x 0.10 x 0.04
Index ranges	$-12 \le h \le 12$<br>$-28 \le k \le 28$<br>$-17 \le l \le 16$	$-17 \le h \le 17$<br>$-22 \le k \le 22$<br>$0 \le l \le 37$
Reflections collected	22829	31048
Independent reflections	6270<br>[R(int) = 0.0803]	31048<br>[R(int) = 0.0921]
Data / restraints / parameters	6270 / 18 / 391	31048 / 582 / 1972
[c]Goodness-of-fit on F^2	0.997	1.041
[a,b]Final R indices [$I>2\sigma(I)$]	R1 = 0.0481<br>wR2 = 0.1091	R1 = 0.0591<br>wR2 = 0.1406
Largest diff. peak / hole, e.Å^{-3}	1.001 / -1.008	2.172 / -1.493

[a] $R = \Sigma||F_o|-|F_c||/\Sigma|F_o|$. [b] $wR = \{\Sigma w(F_o^2-F_c^2)^2/\Sigma w(F_o^2)^2\}^{1/2}$. [c] GOF = $\{\Sigma [w((F_o^2-F_c^2)^2)/(n-p)\}^{1/2}$, where n = number of reflections and p = total number of parameters refined.

Table S2. Selected bond distances (Å) and angles (°) for complexes **3** and **7 x 0.75C$_3$H$_6$O**.

Comp.	C^N				L		Ir-Cp* (centr.)	Bite angle (C-Ir-N)
**3**	Ir(1)-C(10)	2.047(8)	Ir(1)-N(3)	2.083(7)	Ir(1)-N(1)	2.093(7)	1.824	78.3(3)
**7-Ir1**	Ir(1)-C(11)	2.04(1)	Ir(1)-N(3)	2.100(9)	Ir(1)-N(1)	2.083(9)	1.823	79.3(4)
**7-Ir2**	Ir(2)-C(60)	2.091(9)	Ir(2)-N(8)	2.023(9)	Ir(2)-N(6)	2.091(8)	1.823	79.3(4)
**7-Ir3**	Ir(3)-C(89)	2.07(1)	Ir(3)-N(13)	2.043(9)	Ir(3)-N(11)	2.119(9)	1.815	78.5(4)
**7-Ir4**	Ir(4)-C(128)	2.07(1)	Ir(4)-N(18)	2.076(8)	Ir(4)-N(16)	2.084(8)	1.822	78.6(4)

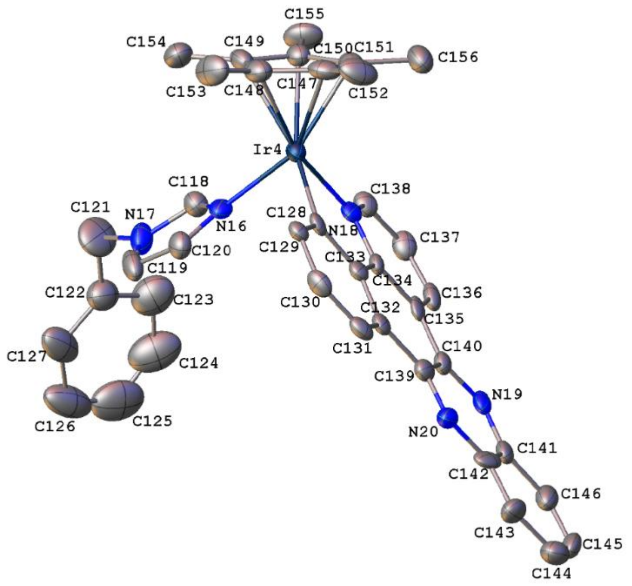

Figure S1. ORTEP diagram of the cation with Ir4 of complex **7**. Ellipsoids are at the 30% probability level. Hydrogen atoms and the BF_4^- anion have been omitted for clarity.

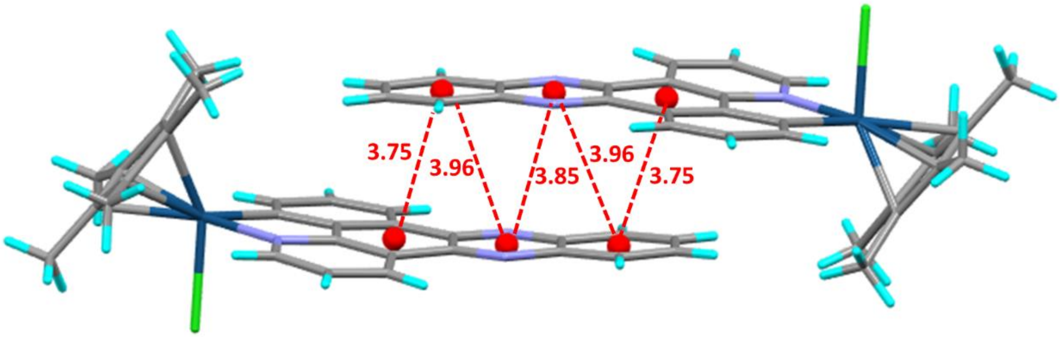

Figure S2. π–π interactions in complex **1** (red, centroid–centroid distances indicated, Å).

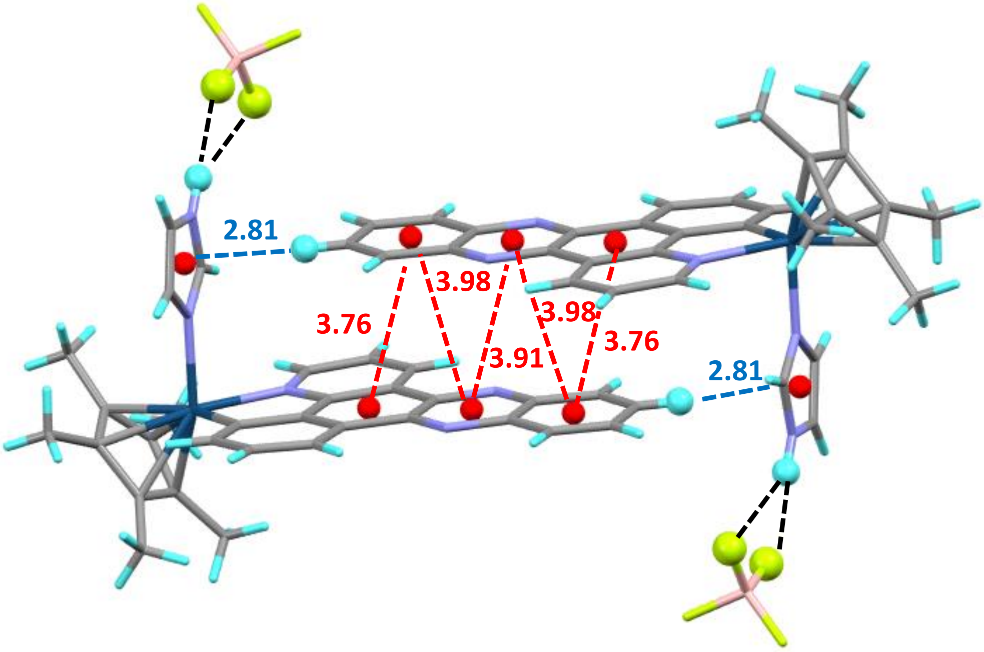

Figure S3. Box-like dimer of complex **3** formed through π–π interactions (red, centroid–centroid distances indicated, Å) and CH–π interactions (blue, centroid–H distance indicated, Å). The hydrogen bonds with the BF_4^- anions are indicated in black. The H and F atoms that participate in H bonds or CH–π interactions are marked as balls.

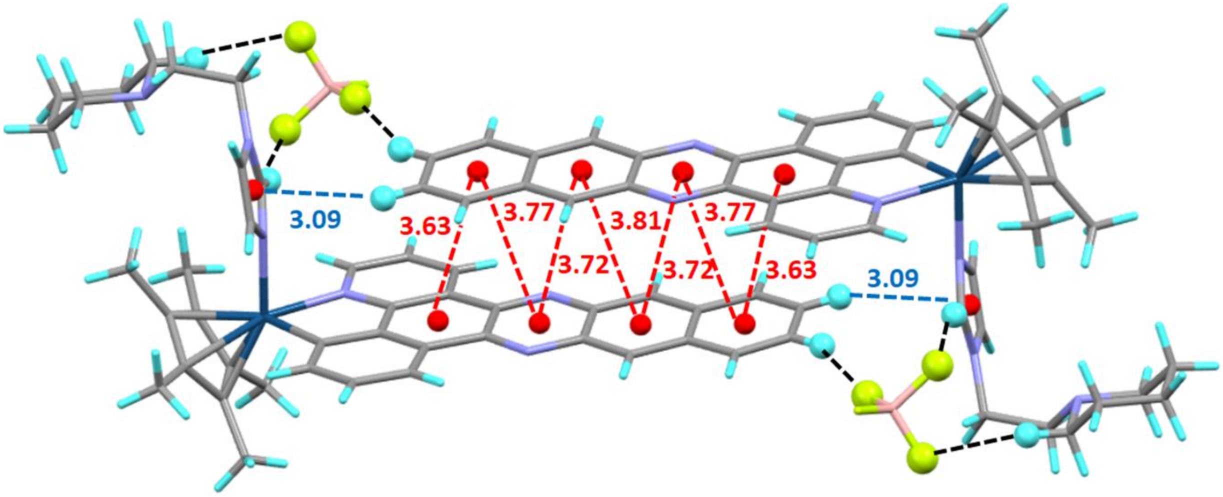

Figure S4. Box-like dimer of complex **6** formed through π-π interactions (red, centroid–centroid distances indicated, Å), CH-π interactions (blue, centroid-H distance indicated, Å) and hydrogen bonds with the BF_4^- anions (black). The H and F atoms that participate in H bonds or CH-π interactions are marked as balls.

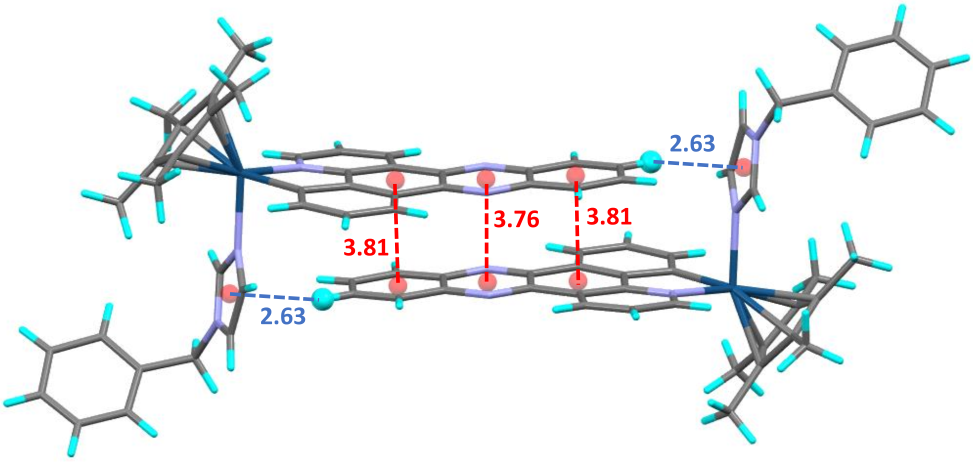

Figure S5. Box-like dimer of complex **7**, of molecules with Ir1, formed through π–π interactions (red, centroid–centroid distances indicated, Å) and CH–π interactions (blue, centroid–H distance indicated, Å). The H atoms that participate in CH–π interactions are marked as balls.

Table S3. Parameters for the π–π in the solid-state structure of complexes **1**, **3**, **6 × 0.75C$_3$H$_6$O** and **7 × 0.75C$_3$H$_6$O**.

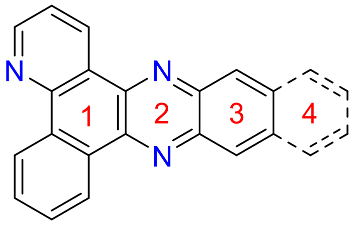

Complex	Rings involved[a]	Ct–Ct (Å)	Ct–pl (Å)	α[b] (°)	β and γ[c] (°)
**1**	3 and 1’ (x2)	3.75	3.51<br>3.36	6.2	20.6<br>26.4
**1**	3 and 2’ (x2)	3.96	3.47<br>3.50	1.7	28.8<br>27.9
**1**	2 and 2’	3.85	3.51	0	24.3
**3**	3 and 1’ (x2)	3.76	3.46<br>3.44	2.4	23.0<br>23.8
**3**	3 and 2’ (x2)	3.98	3.44<br>3.39	1.4	30.2<br>31.6
**3**	2 and 2’	3.71	3.41	0.0	23.2
**6**	4 and 1’ (x2)	3.63	3.38<br>3.25	4.9	21.4<br>26.5
**6**	4 and 2’ (x2)	3.77	3.34<br>3.37	3.4	27.6<br>26.6
**6**	3 and 2’ (x2)	3.72	3.37<br>3.42	1.9	25.1<br>23.2
**6**	3 and 3’	3.81	3.39	0.0	27.2
**7-Ir1**	3 and 1’ (x2)	3.81	3.34<br>3.31	3.5	28.8<br>29.7
**7-Ir1**	2 and 2’	3.76	3.26	0.0	29.9

Ct = centroid, pl = plane. [a] The numbering of the rings is indicated in the figure. For the other ligand involved, primes are used. [b] α is the angle formed between the planes of the two involved rings. [c] β and γ are the angles formed between the Ct–Ct and Ct–pl lines. When α = 0, β = γ.

Table S4. Parameters for the CH–π interactions in the solid-state structure of complexes **3**, **6 × 0.75C$_3$H$_6$O** and **7 × 0.75C$_3$H$_6$O** that leads to the box-like dimers along with the π–π interactions.

Complex	Groups involved	H–Ct (Å)	H–pl (Å)	Ct–H–C (°)	α[a] (°)
**3**	C21H21–Im	3.11	2.78	151.2	26.6
**6**	C32H32–Im	3.09	3.02	145.9	12.2
**7-Ir1**	C27H27–Im	2.63	2.62	165.5	5.0

[a] α is the angle formed between the H–Ct and H–pl lines.

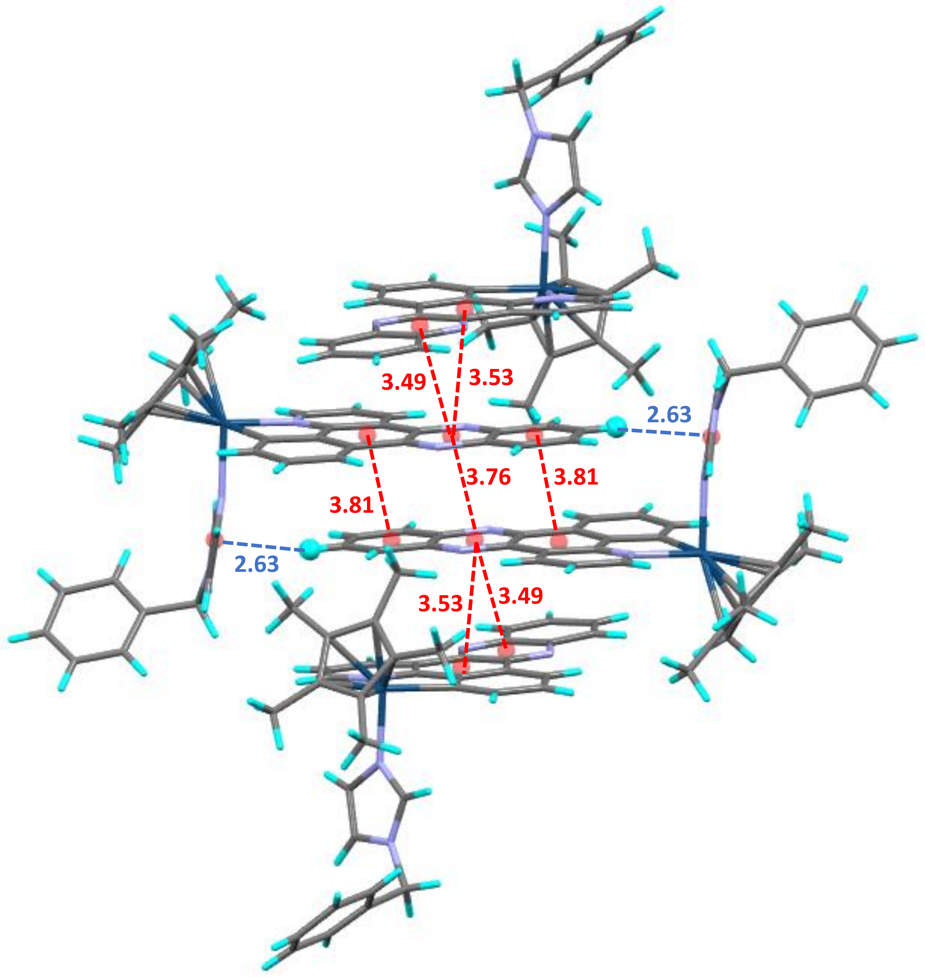

Figure S6. π–π interactions between box-like dimers of complex **7** (red, centroid–centroid distances indicated, Å).

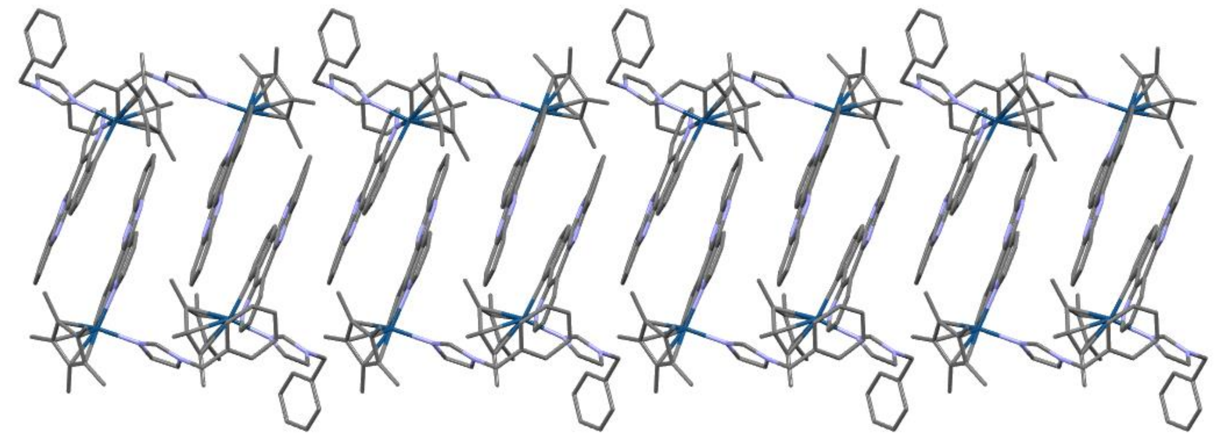

Figure S7. Infinite polymeric chain of complex **7** formed through π–π interactions between box-like dimers (c-axis view).

π–π stacking by NMR spectroscopy

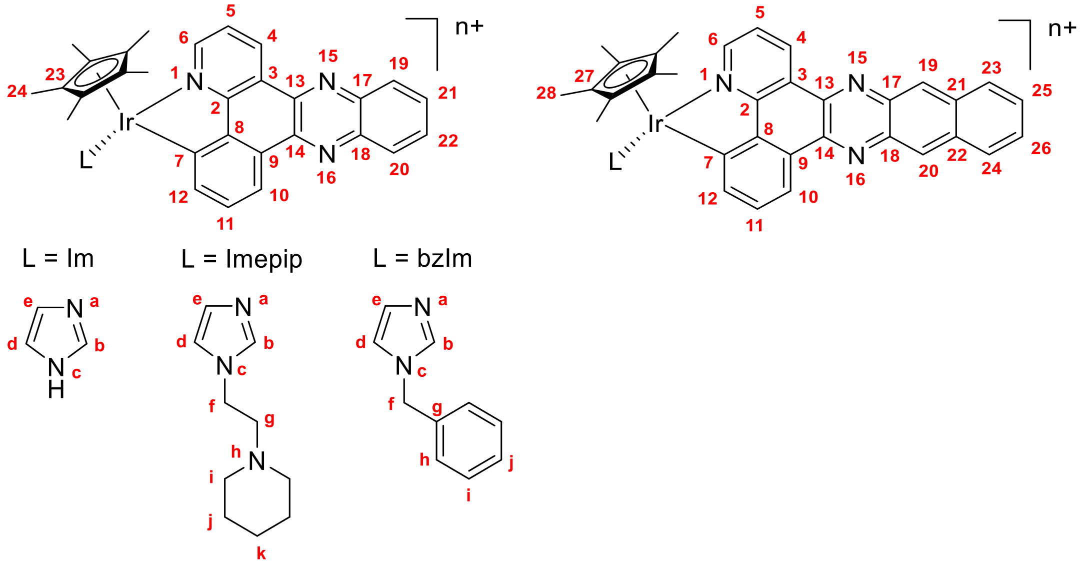

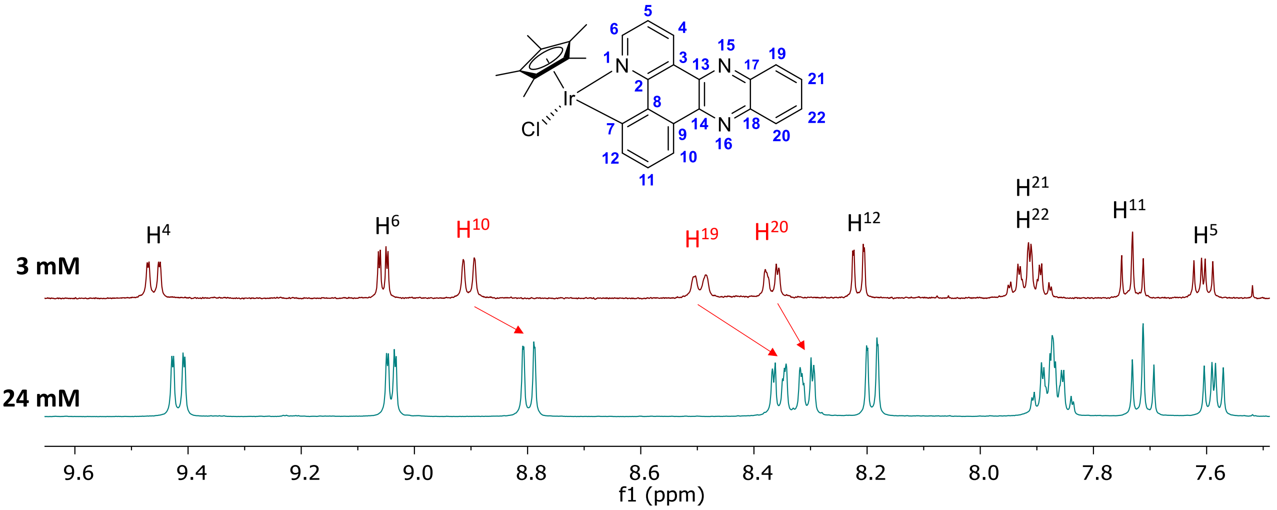

Figure S8. ^{1}H NMR spectra of **1** in $CDCl_3$ at two different concentrations. The protons that suffer higher shifting to lower frequency when the concentration is increased are indicated in red and the arrows reflect this shifting.

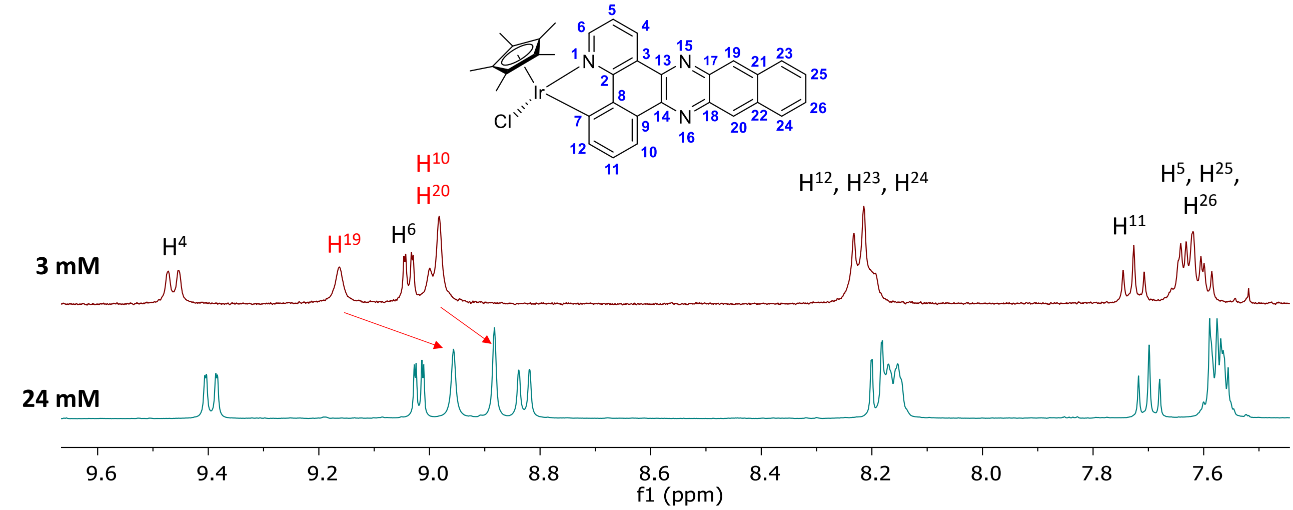

Figure S9. ^{1}H NMR spectra of **2** in $CDCl_3$ at two different concentrations. The protons that suffer higher shifting to lower frequency when the concentration is increased are indicated in red and the arrows reflect this shifting.

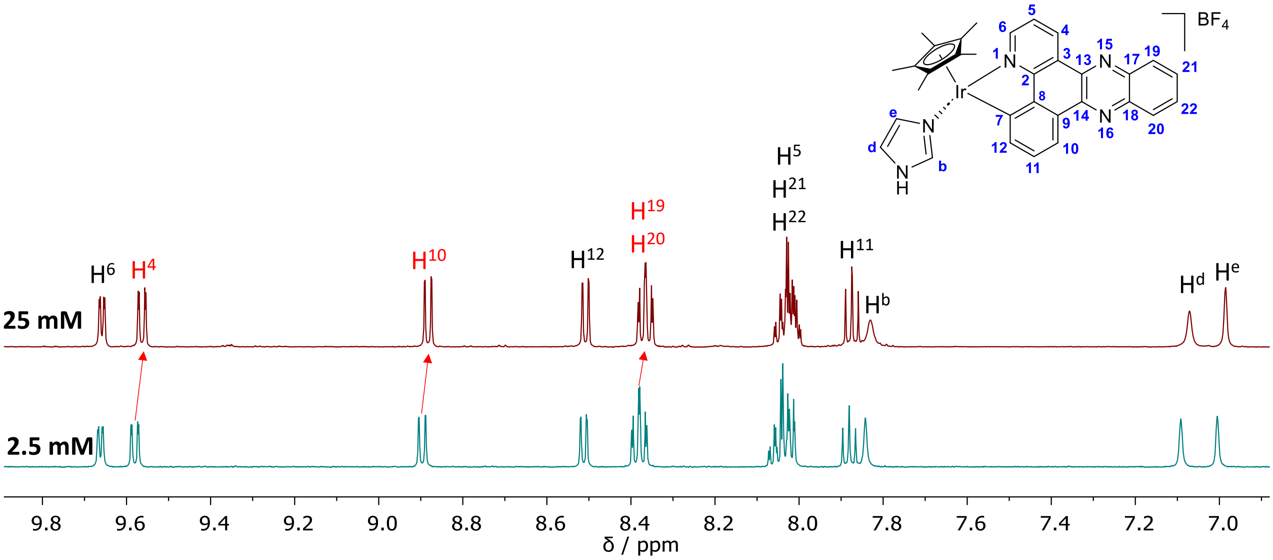

Figure S10. ^{1}H NMR spectra of **3** in acetone-d_6 at two different concentrations. The protons that suffer higher shifting to lower frequency when the concentration is increased are indicated in red and the arrows reflect this shifting

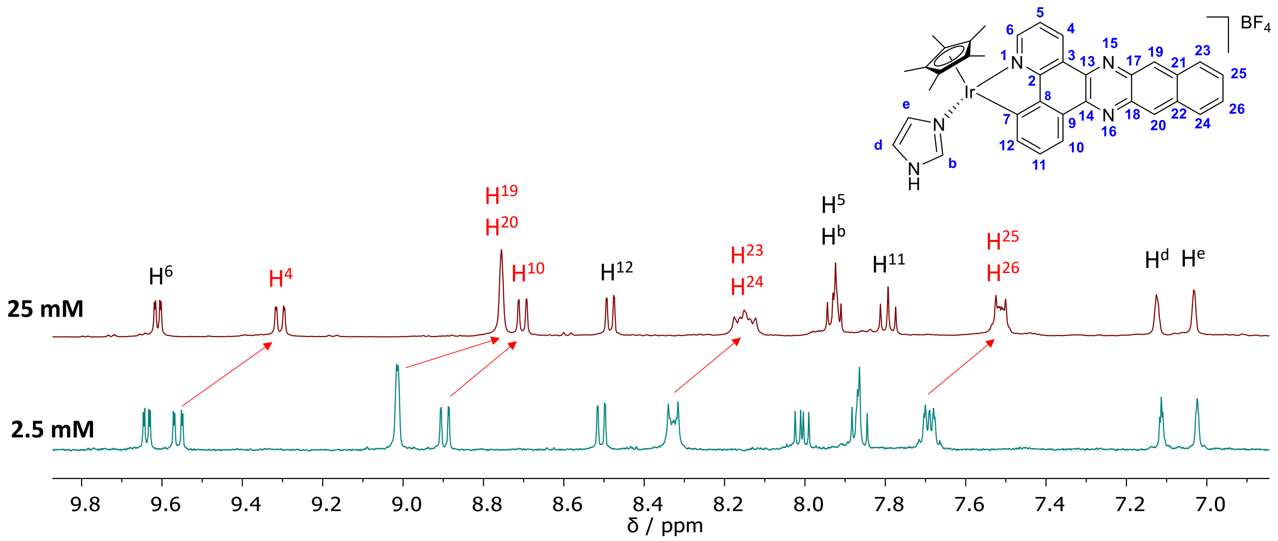

Figure S11. ^{1}H NMR spectra of **4** in acetone-d_6 at two different concentrations. The protons that suffer higher shifting to lower frequency when the concentration is increased are indicated in red and the arrows reflect this shifting.

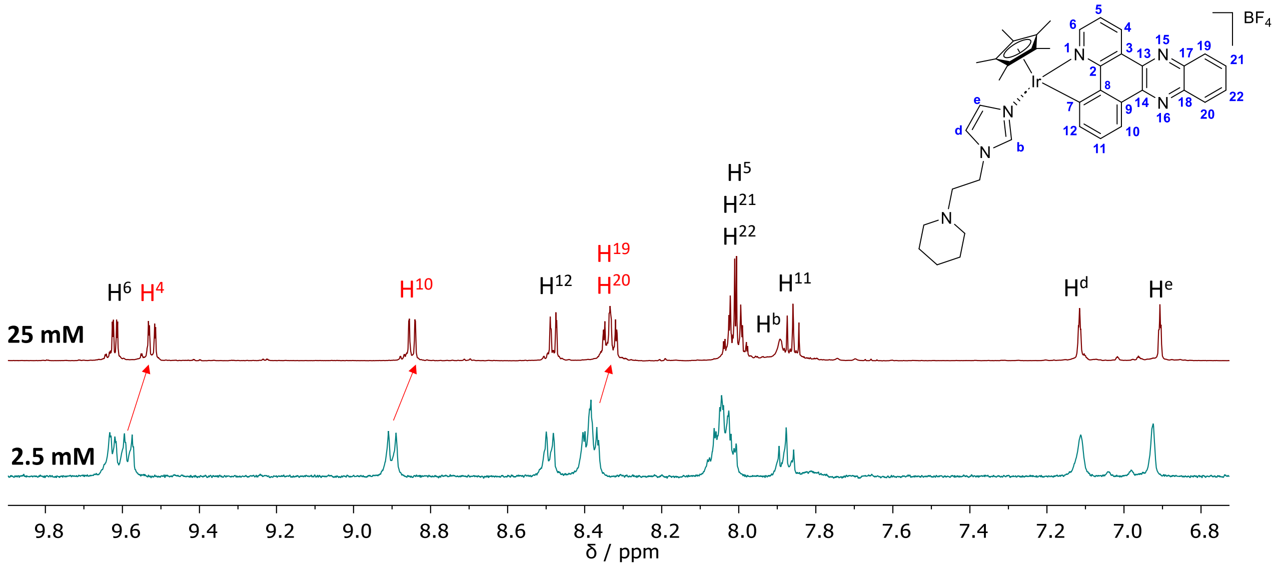

Figure S12. ^{1}H NMR spectra of **5** in acetone-d_6 at two different concentrations. The protons that suffer higher shifting to lower frequency when the concentration is increased are indicated in red and the arrows reflect this shifting.

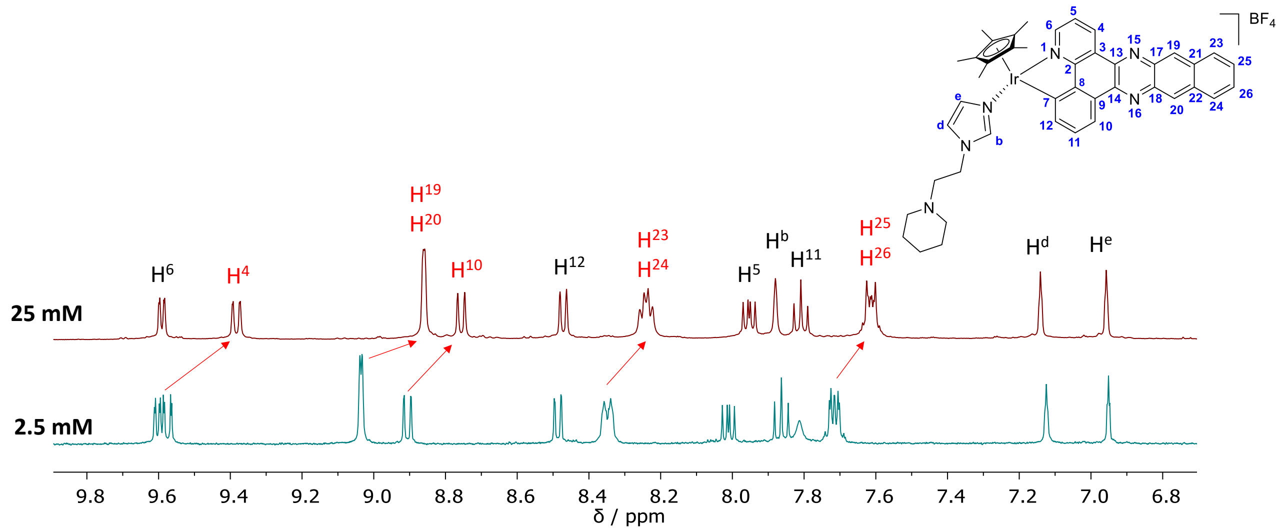

Figure S13. ^{1}H NMR spectra of **6** in acetone-d_6 at two different concentrations. The protons that suffer higher shifting to lower frequency when the concentration is increased are indicated in red and the arrows reflect this shifting.

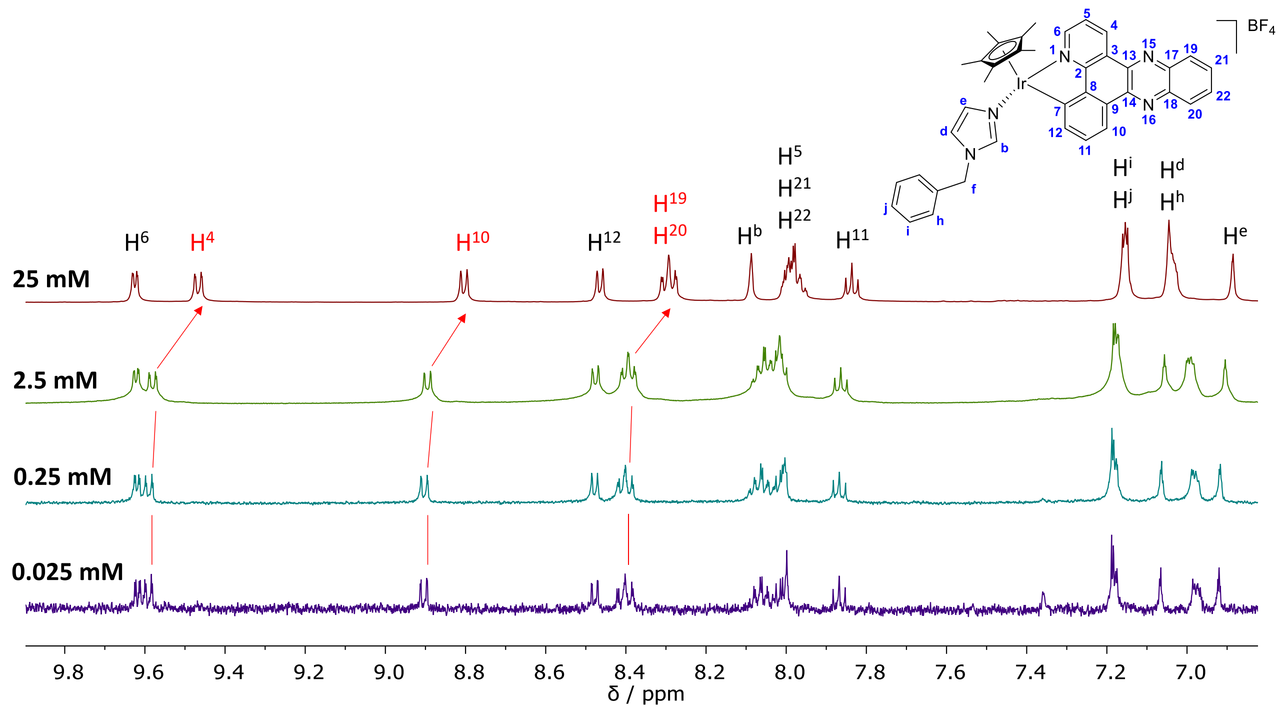

Figure S14. ^{1}H NMR spectra of **7** in acetone-d_6 at two different concentrations. The protons that suffer higher shifting to lower frequency when the concentration is increased are indicated in red and the arrows reflect this shifting.

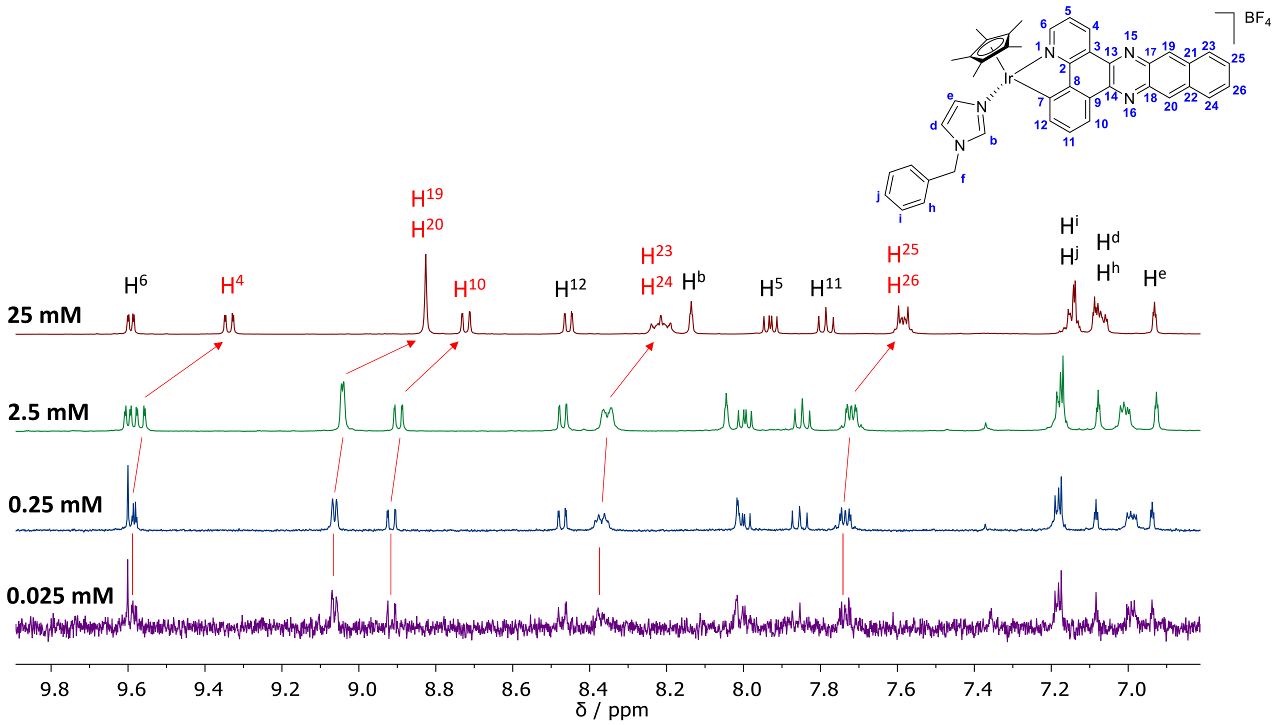

Figure S15. ^{1}H NMR spectra of **8** in acetone-d_6 at two different concentrations. The protons that suffer higher shifting to lower frequency when the concentration is increased are indicated in red and the arrows reflect this shifting.

Table S5. Effect of the concentration on the ^{1}H NMR chemical shifts (δ) of C^N and Cp* ligands of complexes **7** and **8** in acetone-d_6.

					Complex **7**							
**Complex**	**Conc. (M)**	**H^4**	**H^5**	**H^6**	**H^{10}**	**H^{11}**	**H^{12}**	**H^{19}/ H^{20}**	**H^{21}/ H^{22}**	**H^{23}/ H^{24}**	**H^{25}/ H^{26}**	**Cp***
**7**	**25**	9.47	7.98	9.63	8.80	7.84	8.47	8.29	7.98	-	-	1.79
	**2.5**	9.58	8.05	9.62	8.89	7.86	8.48	8.39	8.02	-	-	1.81
	**0.25**	9.59	8.06	9.62	8.90	7.87	8.48	8.40	8.03	-	-	1.82
	**0.025**	9.59	8.06	9.62	8.90	7.87	8.48	8.40	8.03	-	-	1.82
**8**	**25**	9.34	7.93	9.59	8.72	7.79	8.45	8.83	-	8.22	7.59	1.81
	**2.5**	9.57	8.00	9.60	8.90	7.85	8.47	9.04	-	8.35	7.72	1.82
	**0.25**	9.58	8.01	9.60	8.92	7.86	8.47	9.06	-	8.37	7.74	1.83
	**0.025**	9.58	8.01	9.60	8.92	7.86	8.47	9.06	-	8.37	7.74	1.83

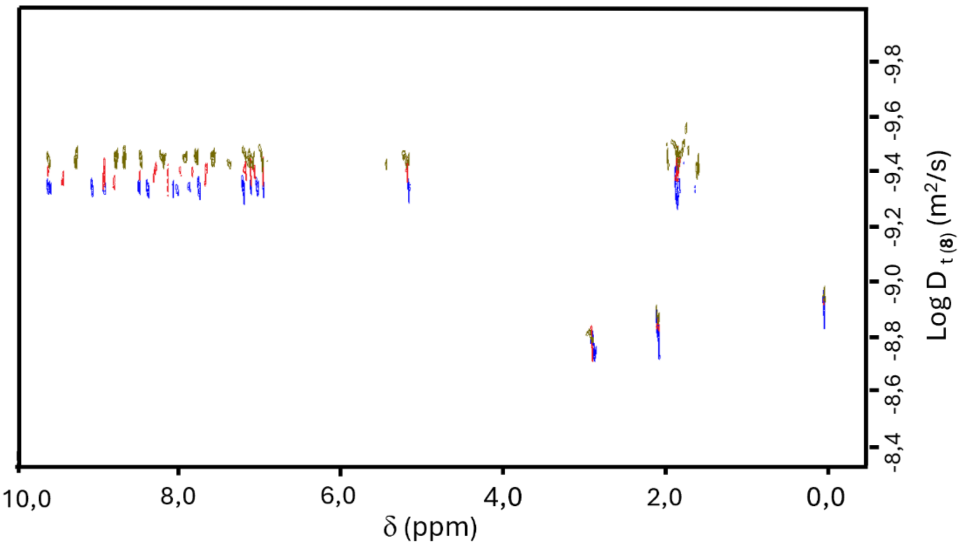

Figure S16. Diffusion coefficients (Log D_t, m^2 s^{-1}) for complex **8** in acetone at different concentrations: 2 mM (blue), 12 mM (red) and 25 mM (green).

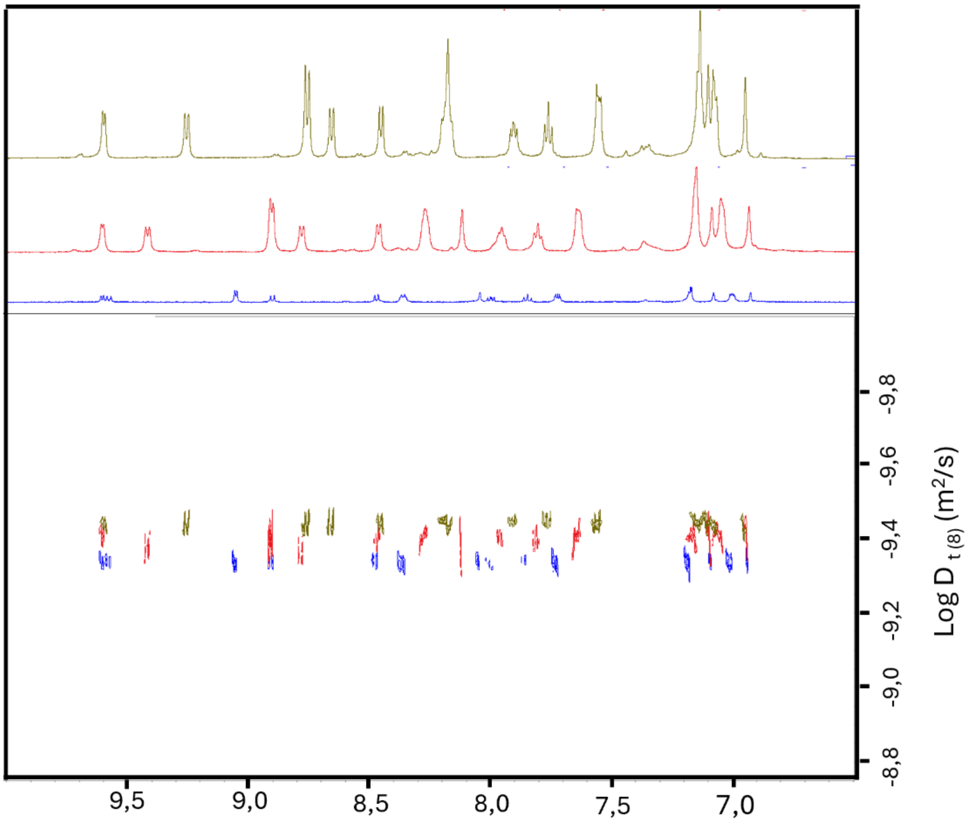

Figure S17. Expansion corresponding to the aromatic region. Diffusion coefficients (Log D_t, m^2 s^{-1}) for complex **8** in acetone at different concentrations: 2 mM (blue), 12 mM (red) and 25 mM (green).

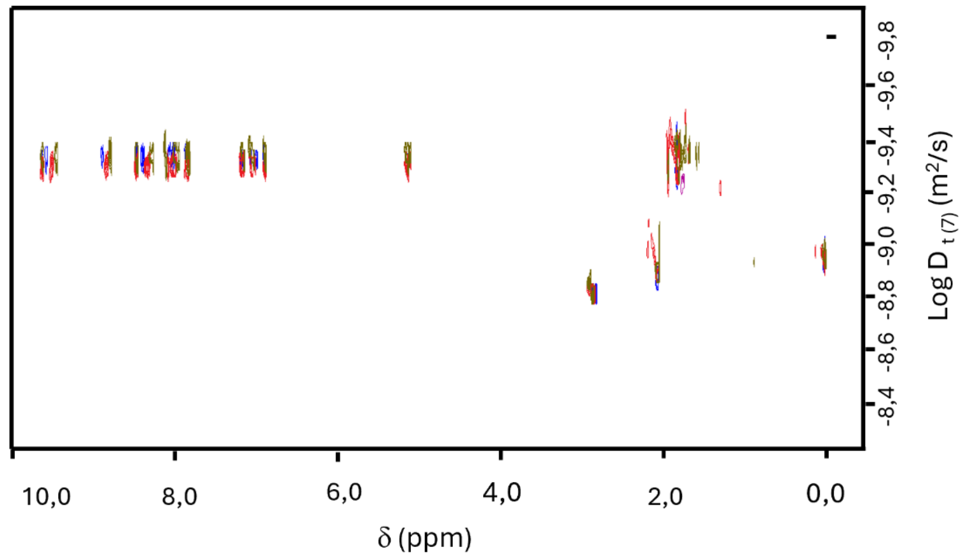

Figure S18. Diffusion coefficients (Log D_t, m^2 s^{-1}) for complex **7** in acetone at different concentrations: 2 mM (blue), 12 mM (red) and 25 mM (green).

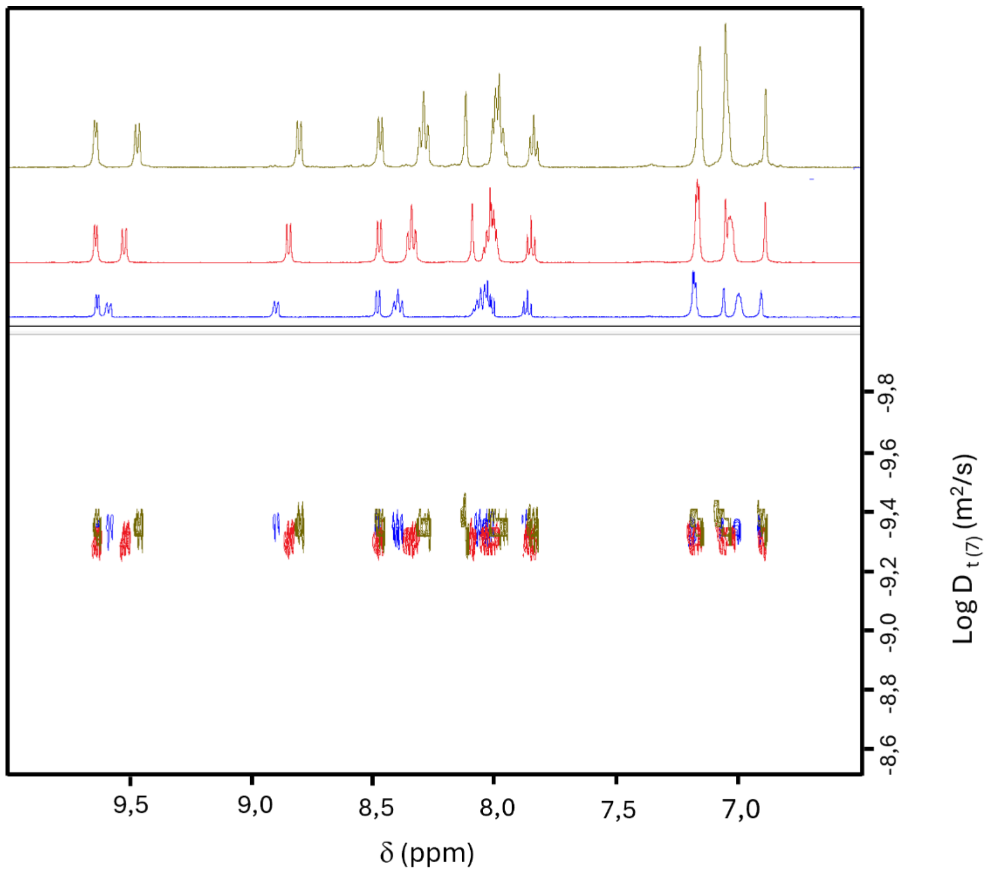

Figure S19. Expansion corresponding to the aromatic region. Diffusion coefficients (Log D_t, m^2 s^{-1}) for complex **7** in acetone at different concentrations: 2 mM (blue), 12 mM (red) and 25 mM (green).

Table S6. Relative diffusion coefficients: $D_{t\ (compound)}$ / $D_{t\ (TMS)}$ for compounds **7** and **8** for the 2 mM, 12mM and 25 mM solutions in acetone, using the Cp* resonance (1.8 ppm) for compounds **7** and **8**.

Entry	Concent. (mM)	$D_{t\ (\mathbf{7})}$/ $D_{t\ (TMS)}$	$D_{t\ (\mathbf{8})}$/ $D_{t\ (TMS)}$
**1**	2	0.36	0.37
**2**	12	0.39	0.33
**3**	25	0.35	0.31

Table S7. Concentration (C, mM) diffusion coefficients (10^{-10} D_t, m^2 s^{-1}) for complexes **7** and **8** and for TMS (10^{-10} D_t, m^2 s^{-1}) in acetone.

		**Compound 7**		**Compound 8**	
**Entry**	**Concent. (mM)**	$D_{t\ (\mathbf{7})}$	D_{TMS}	$D_{t\ (\mathbf{8})}$	D_{TMS}
**1**	2	4.33 (SD: 1.65 · 10^{-3})	11.9 (SD: 4.94 · 10^{-3})	4.54 (SD: 2.22 · 10^{-3})	12.1 (SD: 4.82 · 10^{-3})
**2**	12	4.61 (SD: 2.47 · 10^{-3})	11.9 (SD: 5.67 · 10^{-3})	3.79 (SD: 4.30 · 10^{-3})	11.6 (SD: 3.27 · 10^{-3})
**3**	25	4.13 (SD: 7.06 · 10^{-3})	11.7 (SD: 3.44 · 10^{-3})	3.50 (SD: 3.02 · 10^{-3})	11.4 (SD: 3.23 · 10^{-3})

DLS experiments

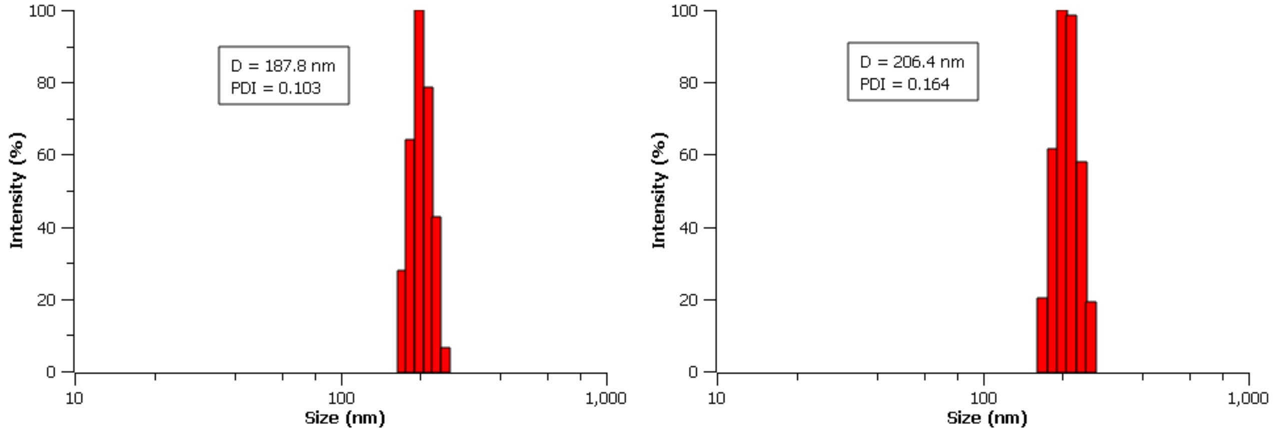

a) b)

Figure S20. DLS of complex **7** at 50 µM (a) and 250 µM (b) in DMSO:H_2O (1:9).

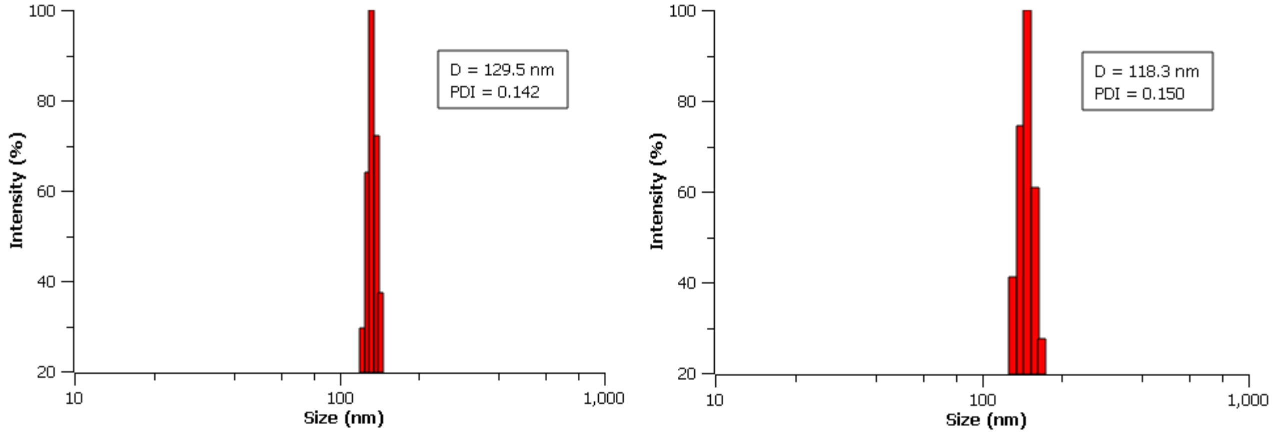

a) b)

Figure S21. DLS of complex **8** at 25 µM (a) and 250 µM (b) in DMSO:H_2O (1:9).

Photophysical properties and IR spectra

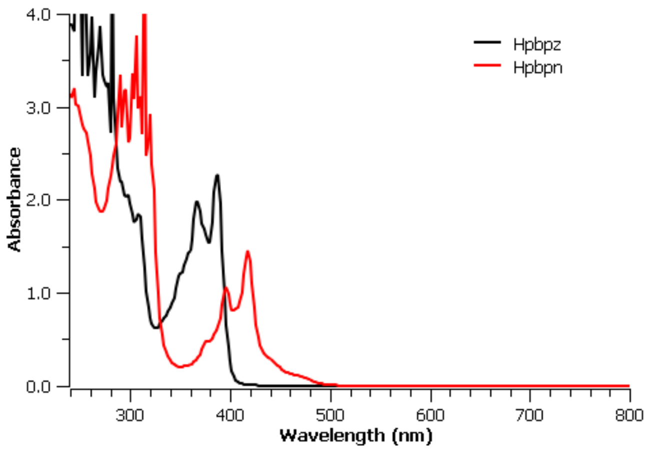

Figure S22. UV-vis absorption spectra of H-pbpz and H-pbpn proligands at 1.0 × 10^{-5} M in degassed acetonitrile.

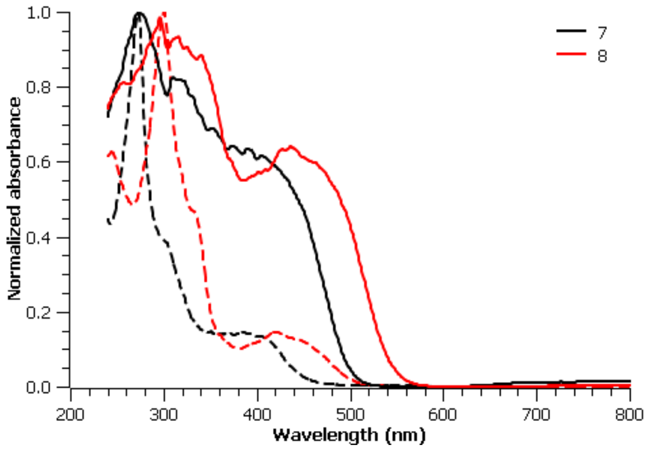

Figure S23. UV-vis absorption spectra of complexes **7** and **8** in the solid state (solid lines) and in solution at 1.0 × 10^{-5} M in degassed acetonitrile (dash lines).

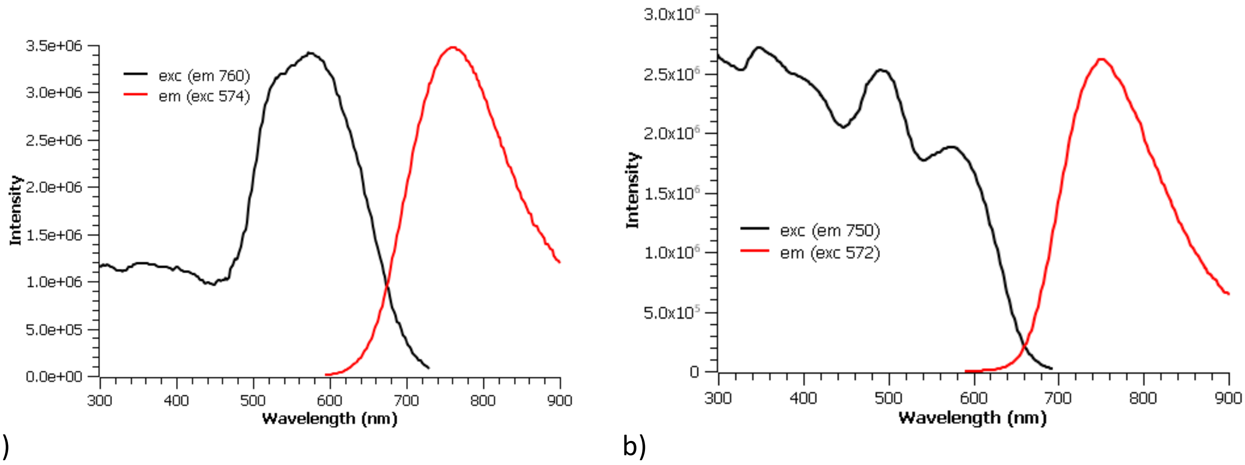

Figure S24. Excitation and emission spectra in the solid state of complex **7** measured at room temperature (a) and at 77 K (b).

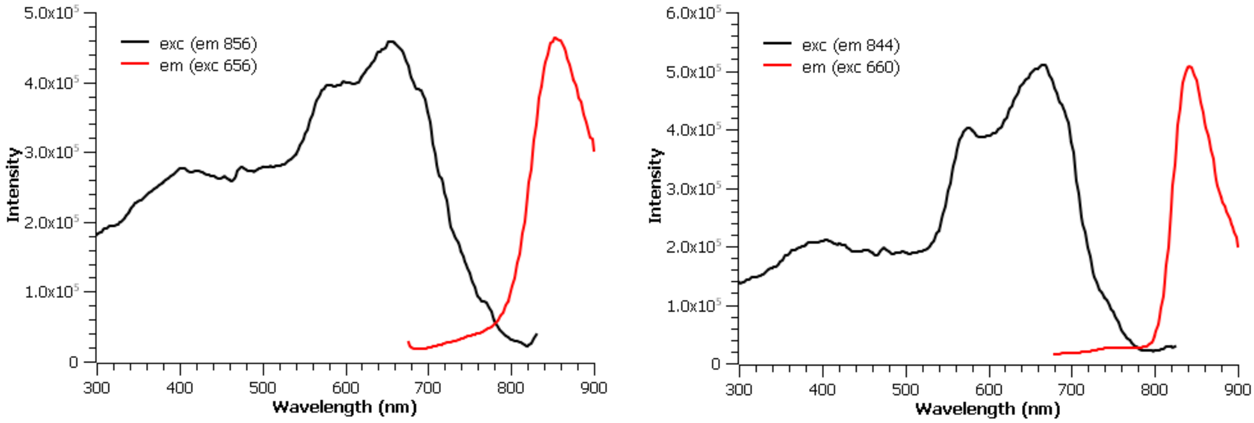

a) b)

Figure S25. Excitation and emission spectra in the solid state of complex **8** measured at room temperature (a) and at 77 K (b).

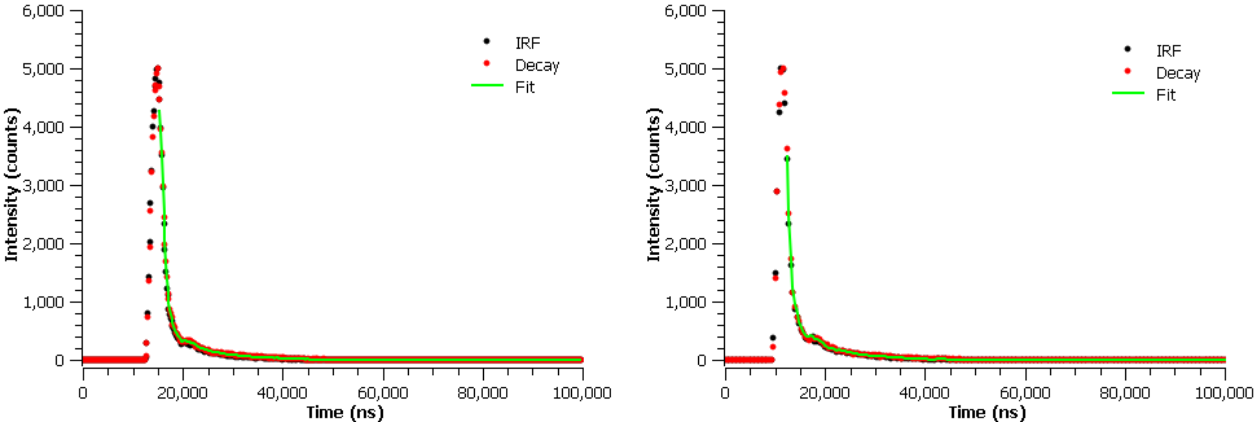

a) b)

Figure S26. Lifetime decays for complexes **7** at 1.0 × 10^{-3} M (a) and **8** at 1.0 × 10^{-5} M (b) in degassed acetonitrile at room temperature: τ = 0.706 ± 0.096 μs (χ^2 = 1.28) (a) and τ = 0.215 ± 0.026 μs (χ^2 = 1.00) (b).

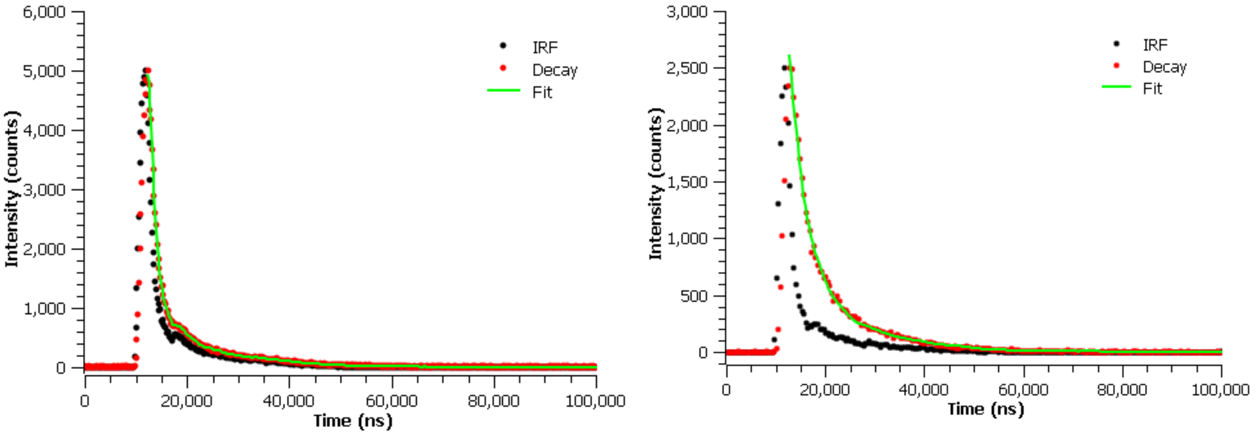

a) b)

Figure S27. Lifetime decay for complex **7** in solid state at room temperature: τ = 0.519 ± 0.028 μs (χ^2 = 1.17) (a) and at 77 K: τ = 2.679 ± 0.050 μs (χ^2 = 1.38) (b).

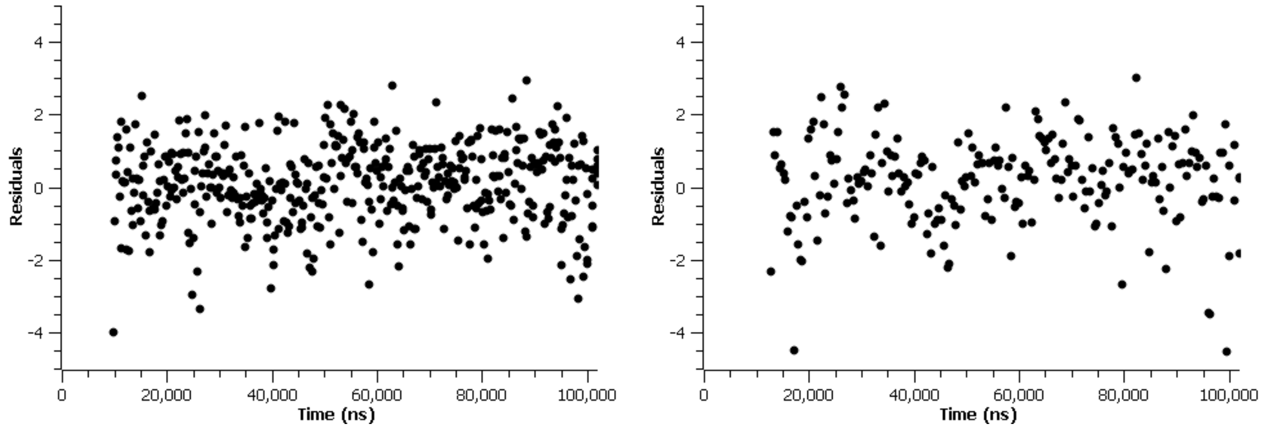

a) b)

Figure S28. Residuals of the lifetime decay fit for complex **7** in solid state at room temperature (a) and at 77 K (b).

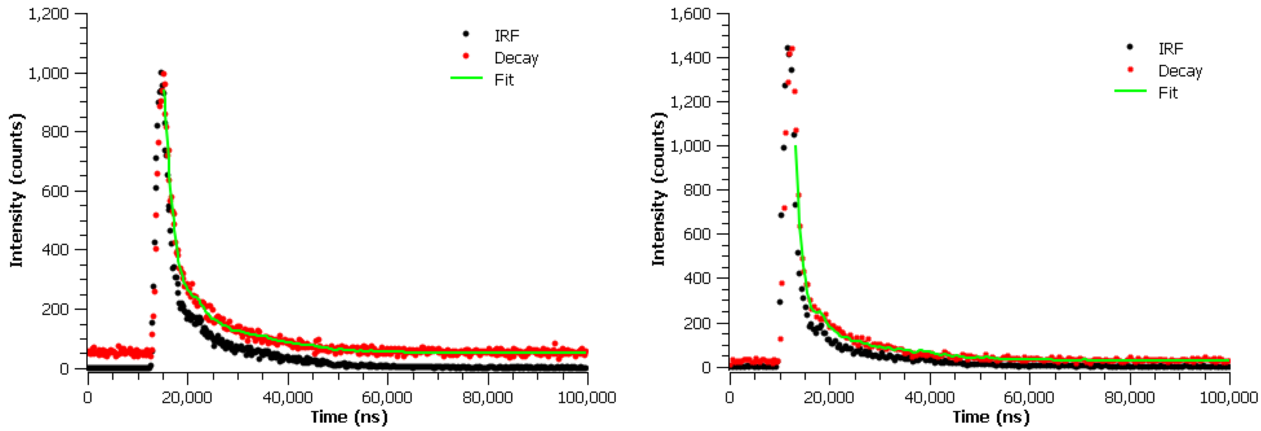

a) b)

Figure S29. Lifetime decay for complex **8** in solid state at room temperature: τ = 0.81 ± 0.05 μs (χ^2 = 1.09) (a) and at 77 K: τ = 0.60 ± 0.05 μs (χ^2 = 1.25) (b).

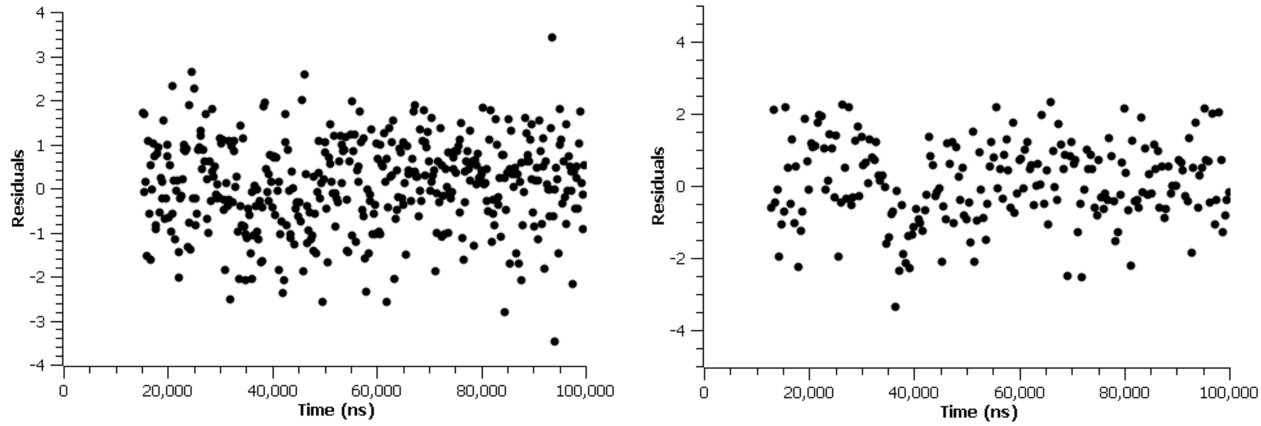

a) b)

Figure S30. Residuals of the lifetime decay fit for complex **8** in solid state at room temperature (a) and at 77 K (b).

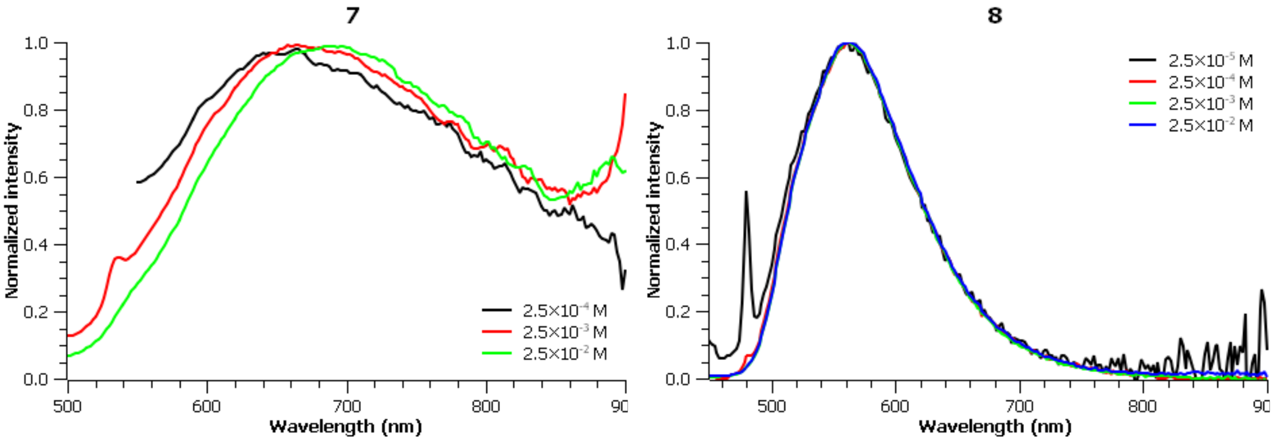

(a) (b)

Figure S31. Normalized emission spectra of complexes **7** (a) and **8** (b) in acetone solution at different concentrations

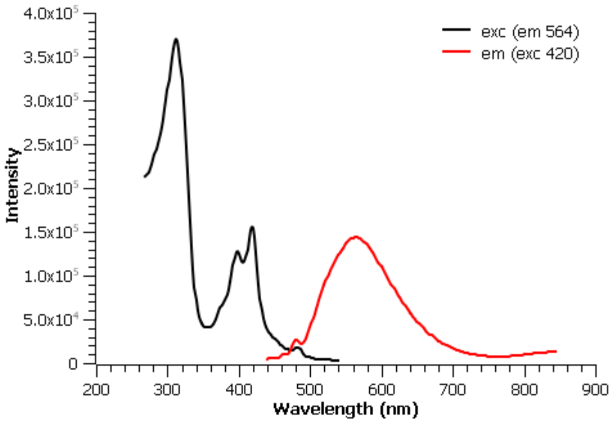

Figure S32. Excitation and emission spectra of complex **8** at 10^{-5} M in degassed acetonitrile at room temperature.

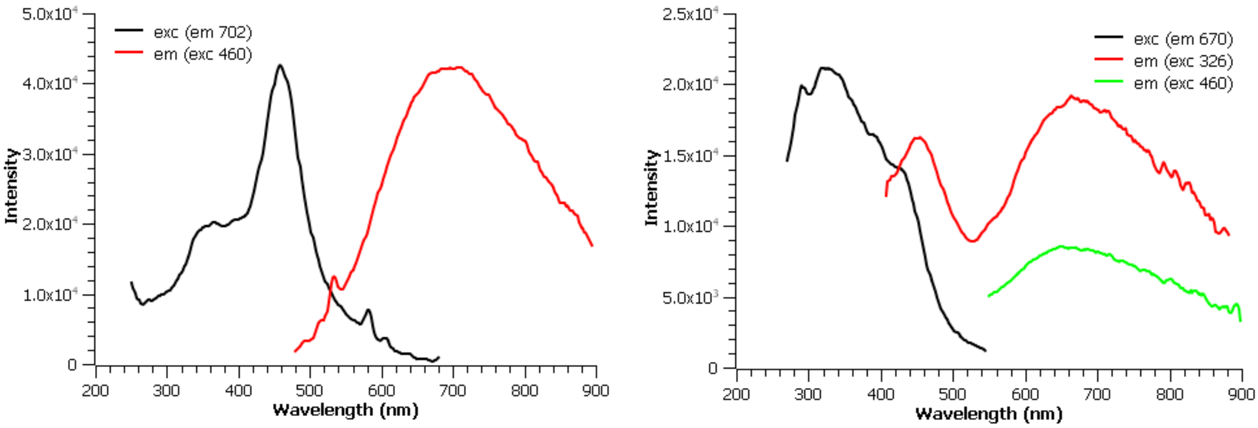

a) b)

Figure S33. Excitation and emission spectra of complex **7** at 10^{-3} M (a) and 10^{-4} M in degassed acetonitrile at room temperature.

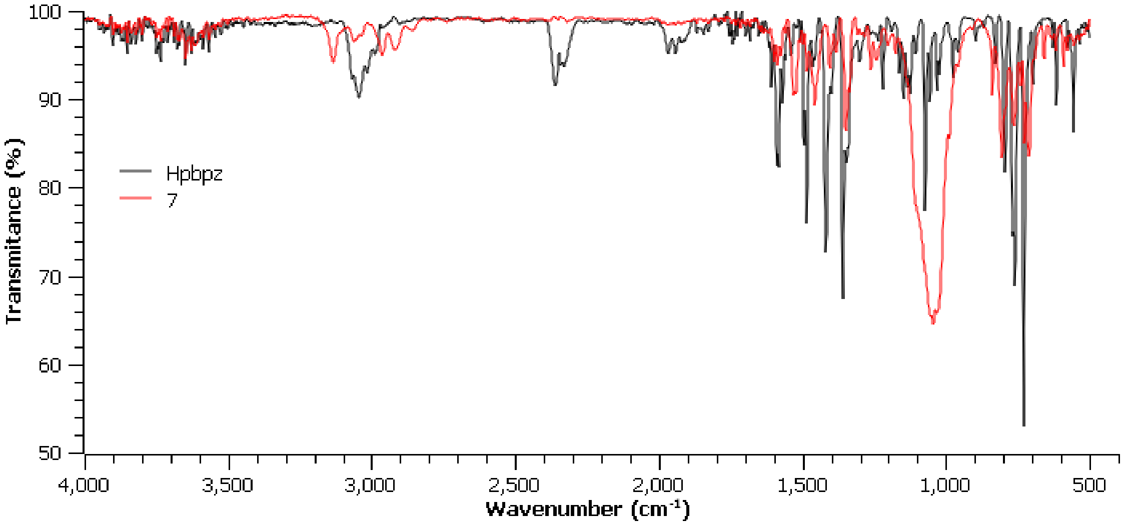

Figure S34. IR spectra of Hpbpz and **7**.

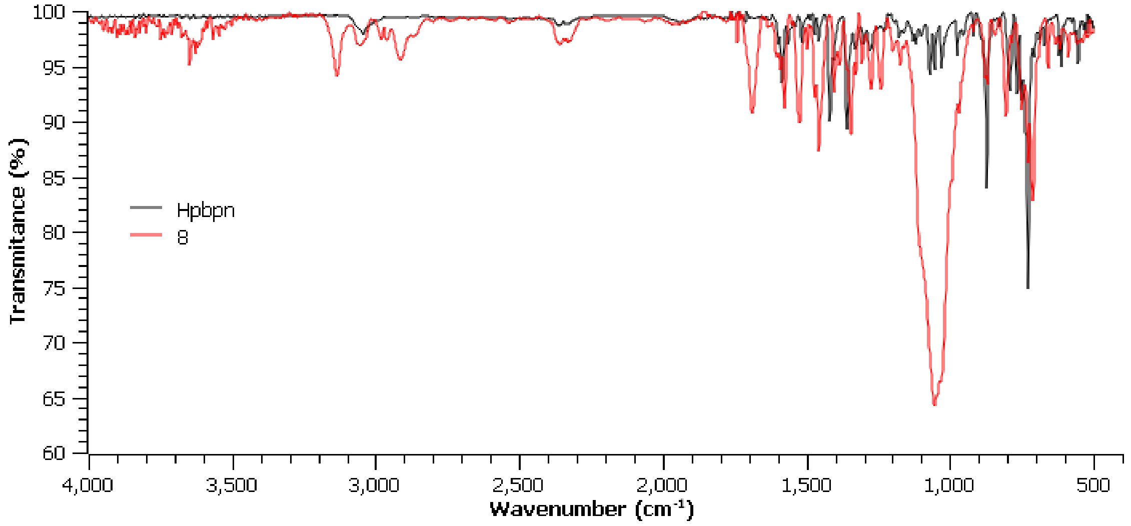

Figure S35. IR spectra of Hpbpn and **8**.

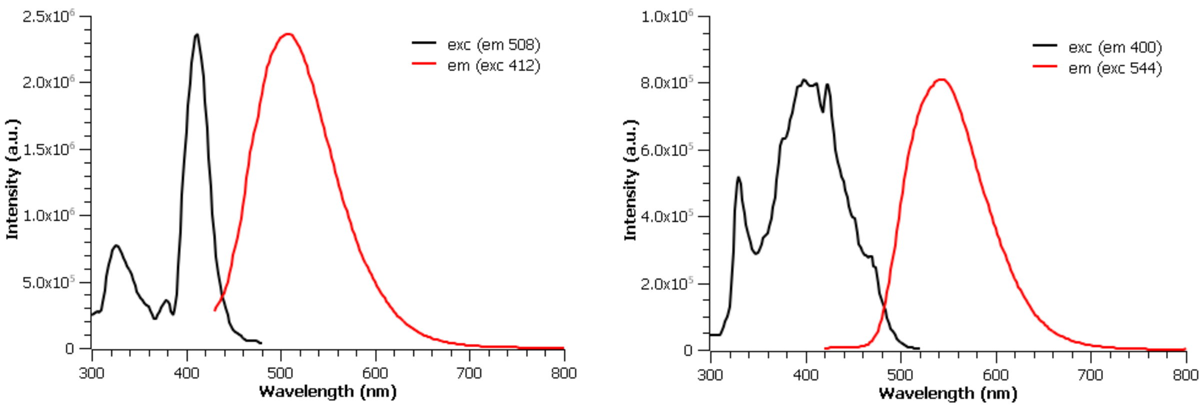

a) b)

Figure S36. Excitation and emission spectra measured in degassed acetonitrile solution at 10^{-5} M at room temperature of proligand H-pbpz (a) and proligand H-pbpn (b).

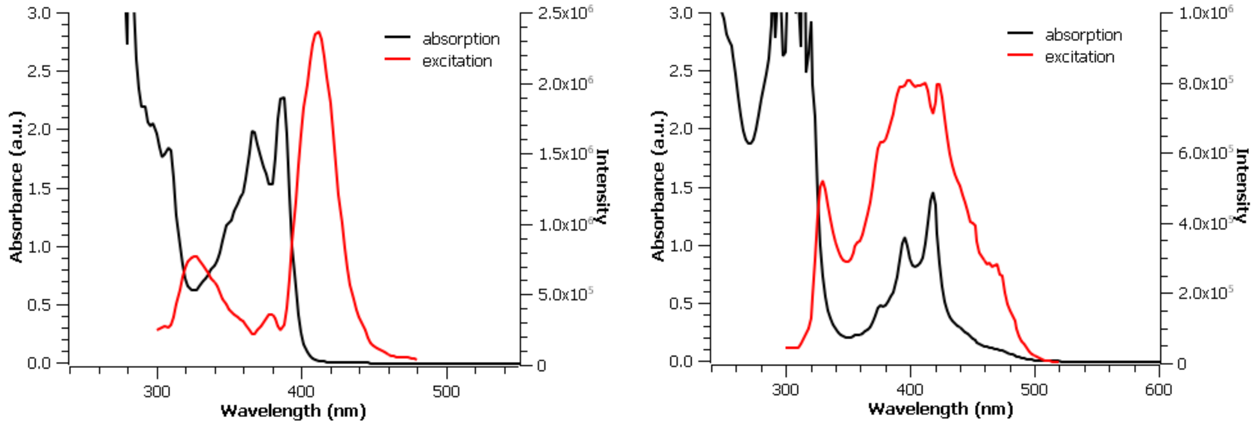

a) b)

Figure S37. Comparison of absorption and excitation spectra of proligand H-pbpz, λ_{exc} = 412 nm; λ_{em} = 508 nm (a) and proligand H-pbpn, λ_{exc} = 400 nm; λ_{em} = 544 nm (b) at 10^{-5} in degassed acetonitrile at room temperature.
